# Spin‐State Modulation of Atomic Iron Sites Enables Efficient CO_2_ Electroreduction in Acid Medium

**DOI:** 10.1002/anie.9239759

**Published:** 2026-04-05

**Authors:** Shanhe Gong, Yanjie Zhai, Qing Xia, Xu Han, Weisong Li, Yiran Ying, Jie Wu, Yingying Zhou, Xiaojie She, Zhaolong Wang, Chundu Wu, Xiaomeng Lv, Xiao Zhang, Shu Ping Lau

**Affiliations:** ^1^ Department of Applied Physics The Hong Kong Polytechnic University Hung Hom Kowloon Hong Kong SAR; ^2^ Department of Mechanical Engineering The Hong Kong Polytechnic University Hung Hom Kowloon Hong Kong SAR; ^3^ Photonics Research Institute The Hong Kong Polytechnic University Hung Hom Kowloon Hong Kong SAR; ^4^ School of Chemistry and Chemical Engineering Jiangsu University Zhenjiang P. R. China; ^5^ School of Agricultural Engineering Jiangsu University Zhenjiang P. R. China; ^6^ State Key Laboratory of Solidification Processing Center for Nano Energy Materials Northwestern Polytechnical University and Shaanxi Joint Laboratory of Graphene (NPU) Xian P. R. China; ^7^ Shenzhen Research Institute The Hong Kong Polytechnic University Shenzhen Guangdong P. R. China

**Keywords:** acidic CO_2_ electroreduction, Fe–N_4_O site, spin‐state transition, PEM reactor, high current density

## Abstract

Electrochemical carbon dioxide (CO_2_) reduction (CO_2_RR) in acidic conditions not only enables high CO_2_ utilization but also reduces the formation of interfacial (bi)carbonate. However, the acidic environment tends to favor the competing hydrogen evolution reaction (HER), which lowers the overall energy efficiency of CO_2_ reduction. Here, we use axial oxygen coordination to tune the spin state of iron‐nitrogen‐carbon sites, shifting from the low‐spin (LS, t_2g_^5^ e_g_^0^) to the medium‐spin (MS, t_2g_^4^ e_g_^1^) state. Experimental results and theoretical simulations show that this medium‐spin structure results in spin‐electron filling of the 𝜎∗ orbital, weakening the interfacial attraction of H_3_O^+^, significantly inhibiting HER, and reducing the *CO desorption energy; thus, CO_2_RR performance in acidic media is greatly improved. The designed Fe−N_4_O structure achieves a mass activity of 76.17 A mg_Fe_^−1^ and CO current densities of approximately 335 mA cm^−2^ in acidic conditions, far exceeding those of Fe−N_4_ (7.86 mA cm^−2^). Meanwhile, the catalyst reaches a high *j*_CO_ of 324.55 mA cm^−2^, 80.97% CO_2_ utilization efficiency, and an energy efficiency of 36.89% in a self‐designed proton‐exchange‐membrane porous‐solid‐electrolyte reactor. This work highlights the spin‐manipulation mechanism for enhancing acidic CO_2_RR performance.

## Graphical abstract

An axial oxygen ligand was added to shift the spin state of the single‐atom Fe site from low to medium spin. This change reduces the interfacial interaction with H_3_O^+^ and decreases the *CO desorption energy, thus enhancing CO_2_RR in acidic media. Consequently, the PEM‐PSE reactor achieves a CO partial current density of 324.55 mA cm^−2^ and a CO_2_ utilization of 80.97%, highlighting its potential for acidic CO_2_ reduction.

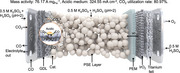

## 1 Introduction

Electrochemical carbon dioxide reduction (CO_2_RR) has gained significant attention as a sustainable method for converting CO2 and integrating it into a carbon‐neutral economic cycle [1, 2]. Among the various products produced by CO_2_RR, carbon monoxide (CO) stands out as a vital feedstock for creating multi‐carbon chemicals and is considered one of the most valuable target products [3, 4]. Extensive research efforts have been dedicated to developing catalysts for CO_2_‐to‐CO electrolysis, achieving industrial‐level activity and selectivity while also reducing the cost of CO production and separation [5, 6, 7]. Furthermore, the CO generated through electrochemical CO_2_ reduction can be further upgraded via electrochemical CO reduction or the Fischer–Tropsch process to produce long‐chain organic chemicals [8, 9]. However, during industrial‐level CO_2_ electroreduction, over 75% of the input CO_2_ reacts with hydroxide (OH^−^), and the main by‐products—carbonate (CO_3_^2−^) or bicarbonate (HCO_3_^−^)—pass through an anion exchange membrane (AEM) to the anode, where they are regenerated to CO_2_, resulting in low carbon efficiency.

In contrast, acidic CO_2_‐to‐CO electrolysis provides an alternative approach to address these challenges [10, 11]. In acidic electrolytes (pH < 3.75), formation of CO_3_^2−^ and HCO_3_^−^ is suppressed [11, 12], enabling higher carbon utilization (>30%) and producing CO that is easier to separate and purify [13, 14]. Additionally, proton exchange membranes (PEMs) can limit carbonate transport to the anode, further decreasing CO_2_ loss [10, 14]. Consequently, acidic environments may be more suitable for CO_2_‐to‐CO electroreduction compared to alkaline or neutral conditions. However, the high concentration of hydronium ions in acid also accelerates the competing hydrogen evolution reaction (HER), which reduces the energy efficiency of CO_2_ reduction [15, 16]. Therefore, these factors make selective CO production in acid challenging, especially at high current densities.

Recent strategies to improve acidic CO_2_ electrolysis focus on adding alkali metal cations to the electrolyte [17, 18]. These cations can alter the interfacial electric field, reducing hydronium migration toward the cathode and thus increasing CO selectivity by decreasing HER [18]. However, at high current densities, this approach struggles to maintain high Faradaic efficiency for CO_2_‐to‐CO conversion in acidic media, with hydrogen becoming the main product. This decline in performance results from two main factors. (1) The strong Coulomb attraction between the negatively charged catalyst surface and hydronium ions at high overpotentials leads to rapid H_3_O^+^ buildup in the electric double layer. This disrupts the interfacial reaction environment and encourages HER [19]. (2) Strong interactions between active sites and reaction products can block site exposure, causing nearby species (such as nitrogen‐containing compounds) to become dominant hydrogen‐evolution sites and trigger severe HER side reactions [20]. Consequently, even catalysts with similar metal single‐atom structures can show significantly different performances [21]. Therefore, effectively preventing the diffusion of interfacial H_3_O^+^ toward active sites at high current densities, while promoting the release of reaction products, will enhance the efficiency of acidic CO_2_ electrolysis and support its industrial application.

Here, we introduce axial oxygen coordination as a strategy to modulate the electronic spin state of conventional Fe–N–C single‐atom sites. Combined experimental and density functional theory (DFT) analyses reveal that introducing an axial oxygen ligand lowers the 3*d* electron density of the Fe center while increasing its effective magnetic moment. This electronic perturbation drives a spin‐state transition from low‐spin in the Fe–N_4_ configuration to medium‐spin in the Fe–N_4_O structure, weakening the interfacial attraction toward H_3_O^+^ and reducing the *CO desorption energy. These changes together facilitate CO_2_ electroreduction in acidic media. The Fe–N_4_O catalyst delivers exceptional performance, reaching industrially relevant current densities of 335 mA cm^−2^ in acidic flow cells, far surpassing that of the Fe–N_4_ counterpart (∼7.9 mA cm^−2^). Furthermore, when integrated into a proton‐exchange membrane (PEM) type porous solid electrolyte reactor, the Fe–N_4_O system achieves a CO partial current density of 324.55 mA cm^−2^ and a carbon utilization efficiency of 80.97%, highlighting its potential for practical applications.

## 2 Results and Discussion

### 2.1 Catalyst Design and Preparation

To achieve high energy efficiency in CO_2_RR in acidic media, the first step is to strategically design the catalytic site to suppress the competing HER reaction at high currents. We selected single‐atom Fe sites for this study due to their well‐defined Fe–N_4_–C coordination and the tunable spin state of the central Fe atom, which enables precise control over catalytic behavior. Previous studies have shown that the Fe 3*d* orbitals undergo dynamic electron occupancy and vacancy during electrochemical operation, facilitating charge transfer and promoting activity. Structurally, the Fe–N_4_ motif typically adopts a square planar configuration with *D_4h_* symmetry [22, 23]. In this geometry, the 3*d* orbitals split into distinct energy levels: *d_x_^2^_–y_^2^*, *d_z_^2^*, and *d_xy_* (with simple degeneracy), and *d_yz_*, *d_xz_* (with double degeneracy). The distribution of spin electrons among these orbitals allows for multiple spin configurations of the Fe center. Several pioneering studies have demonstrated that the spin state plays a crucial role in modulating the binding strength between the oxygen intermediate's *p* orbitals and the Fe *d* orbitals, significantly influencing reaction pathways. In the case of oxygen reduction reaction (ORR), [24] certain spin configurations facilitate the release of *OH intermediates, thereby accelerating reaction kinetics.

Inspired by these findings, it is expected that tuning the spin configuration of Fe atoms by adding axial nonmetallic elements could adjust the interaction strength between Fe sites and H_2_O, thereby suppressing, under high current densities, the diffusion of H^+^ ions toward the cathode interface through the interfacial H_2_O network and consequently reducing the hydrogen evolution reaction. Meanwhile, the spin‐optimized Fe sites might weaken their interaction with *CO, which would facilitate CO desorption and re‐expose Fe active sites, potentially preventing HER on N sites and decreasing H_2_ by‐product formation.

### 2.2 Synthesis and Characterization of Fe‐N_4_O Catalyst

**FIGURE 1:**
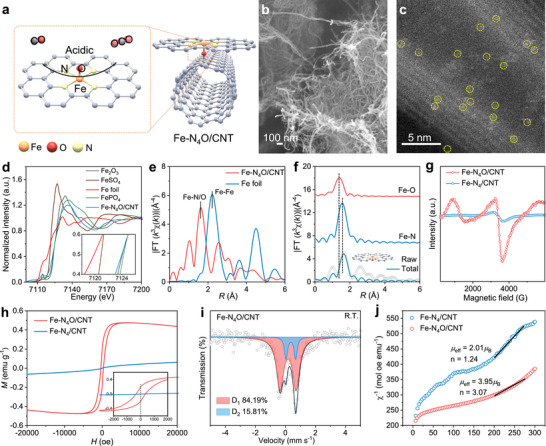
Synthesis and characterization of the Fe‐N_4_O/CNT catalyst. (a) Mechanism diagram of the Fe‐N_4_O/CNT catalyst. (b) SEM image of the Fe‐N_4_O/CNT catalyst. (c) AC HAADF‐STEM image of the Fe‐N_4_O/CNT catalyst. (d) Experimental Fe K‐edge XANES spectra of Fe‐N_4_O/CNT, Fe foil, Fe_2_O_3_, FeSO_4,_ and FePO_4_, and (e) Fourier‐transformed magnitudes of the Fe K‐edge EXAFS signals for both Fe‐N_4_O/CNT and Fe foil. (f) Fe K‐edge EXAFS analysis of Fe‐N_4_O/CNT in R space. Curves from top to bottom are the Fe–O (red) and Fe–N (blue) backscattering signals included in the fit (cyan) superimposed on the experimental signal (black). The inset in (f) shows a structure of a Fe‐N_4_O moiety derived from the EXAFS results, where the orange, red, yellow, and grey spheres represent Fe, O, N, and C, respectively. (g) The EPR spectra of Fe‐N_4_O/CNT and Fe‐N_4_/CNT. (h) Magnetic hysteresis loops of Fe‐N_4_O/CNT and Fe‐N_4_/CNT at room temperature. (i) The room temperature ^57^Fe Mössbauer spectrum of Fe‐N_4_O/CNT. (j) 𝜒^−1^‐T plots of Fe‐N_4_O/CNT and Fe‐N_4_/CNT.

To synthesize the Fe‐N_4_O catalyst, commercial protoporphyrin IX iron chloride (FePPCl, Figure S1) was first refluxed with hydroxyl‐functionalized carbon nanotubes (CNT‐OH) in ethanol, with triethylamine added as a base. During this process, a covalent bond forms between the Fe and a surface oxygen atom, producing hydrochloric acid (HCl), which is then removed by triethylamine through the formation of triethylamine hydrochloride. The resulting hybrid material, FePP‐O‐CNT, was thoroughly washed with ethanol to remove impurities and loosely bound porphyrins. The FePP‐O‐CNT was then carbonized in an argon atmosphere at 800°C, producing the catalyst called Fe‐N_4_O/CNT (Figure 1a). The Fe‐N_4_/CNT was prepared using a similar method, but with iron phthalocyanine (Fe(III)Pc) and multi‐walled carbon nanotubes (CNT) as starting materials.

To further clarify the morphological and structural features of Fe‐N_4_O/CNT, scanning electron microscopy (SEM) analysis was performed, revealing that the material retains the one‐dimensional structure of the original carbon nanotubes (CNTs) without aggregation of FePPCl residues, as shown in Figure 1b. Notably, aberration‐corrected high‐angle annular dark‐field scanning transmission electron microscopy (HAADF‐STEM) at atomic resolution identifies individual dispersed Fe atoms on the CNT surface as bright dots (Figure 1c). This confirms the successful formation of single iron atoms on the CNTs, consistent with x‐ray diffraction (XRD) results (Figure S2c). Elemental mappings from energy‐dispersive x‐ray (EDX) spectroscopy further support the even distribution of isolated Fe sites across the CNTs, along with uniformly dispersed nitrogen and oxygen elements (Figure S3). Fe‐N_4_/CNT exhibits similar morphological features to Fe‐N_4_O/CNT, as seen in XRD (Figure S2b), SEM, and transmission electron microscopy (TEM) analyses (Figure S4b). Additionally, inductively coupled plasma optical emission spectroscopy (ICP‐OES) results indicate that the active Fe site loading in Fe‐N_4_O/CNT and Fe‐N_4_/CNT is 0.44 wt% and 0.50 wt%, respectively (Table S1). Increasing the amount of FePPCl added led to the formation of Fe nanoparticles (NPs) after pyrolysis (Figure S5).

Additional measurements were conducted to verify successful synthesis and to better understand the electrocatalytic properties of Fe‐N_4_O/CNT and Fe‐N_4_/CNT. The electronic structures of Fe‐N_4_O/CNT, Fe‐N_4_/CNT, and FePPCl were analyzed using x‐ray photoelectron spectroscopy (XPS). Importantly, no Cl signal was detected in Fe‐N_4_O/CNT (Figure S6f), unlike FePPCl, which displayed a distinct Cl 2*p* peak at about 198.2 eV (Figure S7e), indicating that the axial element in Fe was successfully replaced. It is also notable that Fe remains in the +III oxidation state on Fe‐N_4_O/CNT, as shown by a Fe 2*p*_3/2_ signal at 712.03 eV (Figure S6c), close to FePPCl's value (711.84 eV, Figure S7c) and that of Fe‐N_4_/CNT (+III, 713.52 eV, Figure S8c). [25, 26] Furthermore, the presence of Fe‐O species in Fe‐N_4_O/CNT at 531 eV further confirms the contribution of axial oxygen (Figure S6e).

The local geometric and electronic structure of the Fe atom in Fe‐N_4_O/CNT was further analyzed using x‐ray absorption spectroscopy (XAS, Figure 1d). The XANES spectra of Fe‐N_4_O/CNT differed from those of Fe foil, indicating a modified electronic structure and a higher valence state (Figure S9), which agrees with XPS analysis. The Fourier‐transformed k^3^‐weighted EXAFS spectrum of physically mixed Fe‐N_4_O/CNT showed a main peak at 1.61 Å, representing the Fe‐N/O contribution. No Fe‐Fe bonds were detected in Fe‐N_4_O/CNT, confirming the absence of iron nanoparticles and clusters. Wavelet transform (WT) EXAFS analysis was performed in k‐space to identify backscattering atoms, due to the limited resolution of Fe‐N(O) in R‐space. The strongest scattering path for Fe‐N_4_O/CNT appears at 5.8 Å^−1^ (Figure S10), which is different from the Fe‐Fe path in Fe foil (8.1 Å^−1^), ruling out Fe‐Fe bonds in the Fe‐N_4_O/CNT sample. A least‐squares method was employed to study the coordination environment of Fe atoms in Fe‐N_4_O/CNT. Based on XPS results, the EXAFS spectrum of Fe‐N4O/CNT was fitted considering backscattering paths to Fe–N and Fe–O. The best fit shows that the main peak at 1.61 Å originates from Fe–N and Fe–O first‐shell coordination. The coordination numbers of N and O atoms in the first shell around Fe are estimated to be approximately 4 and 1, respectively, indicating a square‐pyramidal structure for the Fe─N/O bonding.

To further explore how axial oxygen affects the spin states of Fe species, magnetic hysteresis loops were recorded at room temperature. As shown in Figure 1g, electron paramagnetic resonance (EPR) spectroscopy results indicate that adding an axial oxygen ligand increases the number of unpaired electrons, thus changing the spin state of single atom Fe from low spin to medium spin. Figure 1h shows that the saturation magnetization rises from approximately 0.06 to 0.47 emu g^−1^. An enlarged view of the curve near *H* = 0 is provided in the inset of Figure 1h. Fe‐N_4_O/CNT exhibits a coercive magnetic field (Hc) of 349 Oe and a residual magnetization (Mr) of about 0.19 emu g^−1^, higher than those of Fe‐N_4_/CNT. These results confirm that the addition of axial oxygen influences the spin state of Fe by increasing the number of unpaired electrons.

To gain insight into the electron spin affected by axial ligands, a detailed analysis using ^57^Fe Mössbauer spectroscopy is provided. ^57^Fe Mössbauer spectroscopy effectively identifies the coordination properties and spin states of Fe species. The Mössbauer spectra of Fe‐N_4_O/CNT are well deconvoluted into two doublets, D_1_ and D_2_, which are assigned to the intermediate‐spin and low‐spin states of Fe, respectively (Figure 1i; Table S2), [24, 27, 28], demonstrating the presence of medium spin states in Fe‐N_4_O/CNT.

To better understand how the electron spin configuration affects the catalysts, zero‐field cooling temperature‐dependent (ZFC‐T) magnetic susceptibility (𝜒^m^) measurements were conducted (Figure S11). By fitting the 𝜒^−1^ vs. T curve (Figure 1j), the effective magnetic moments (*µ*_eff_) of Fe‐N_4_O/CNT and Fe‐N_4_/CNT were found to be 3.95 *µ*_B_ and 2.01 *µ*_B_, respectively. Additionally, the number of unpaired electrons in Fe‐N_4_O/CNT is confirmed to be approximately 3, compared to about 1 in Fe‐N_4_/CNT. Because of the larger effective magnetic moment and greater number of unpaired *d* electrons, the interaction between Fe‐N_4_ and axial oxygen causes a change in the electronic structure of the Fe single atoms, shifting the spin state from low spin (LS, t_2g_^5^e_g_^0^) to medium spin (MS, t_2g_^4^e_g_^1^), which supports our hypothesis.

### 2.3 Electrochemical CO_2_ Reduction Performance in the Flow Cell

**FIGURE 2:**
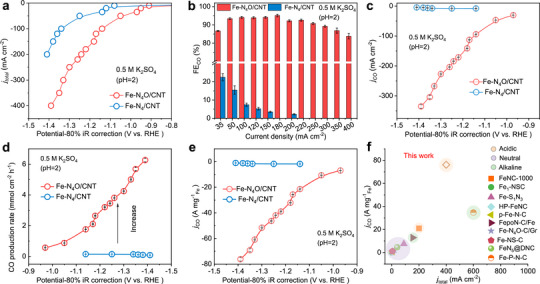
Electrochemical CO_2_ reduction test in the flow cell. (a) *j_total_*, (b) *FE_CO_*, (c) *j_CO_*, (d) CO production rate, and (e) CO mass activity of Fe‐N_4_O/CNT and Fe‐N_4_/CNT. (f) The high CO mass activity of Fe‐N_4_O/CNT compared to reported Fe‐based single‐atom catalysts. The error bars in b, c, d, and e indicate the standard deviation of three independent measurements. Data are shown as mean values ± s.d.

The structural characterization of the catalyst matches our initial goals, leading to the integration of gas diffusion electrodes (GDEs) with Fe‐N_4_O/CNT and Fe‐N_4_/CNT electrocatalysts (Figure S12) to assess their performance at industrially relevant current densities and selectivity in acidic electrolytes (0.5 M K_2_SO_4_ + H_2_SO_4_, pH = 2). Electrochemical measurements were first performed in a flow cell. Gas‐phase products were monitored online using gas chromatography (GC), while liquid‐phase products were measured by ^1^H nuclear magnetic resonance (NMR) spectroscopy. Compared to Fe‐N_4_/CNT, the Fe‐N_4_O/CNT catalyst showed significantly higher activity, as confirmed by linear sweep voltammetry (LSV). As shown in Figure 2a, Fe‐N_4_O/CNT reaches a current density of 200 mA cm^−2^ at around −1.24 V versus RHE, outperforming Fe‐N_4_/CNT (−1.41 V), indicating that adding axial oxygen ligands improves CO_2_RR performance at Fe sites.

In Figure 2b, Fe‐N_4_/CNT shows a lower CO Faraday efficiency (FE_CO_) of 25% across a current density range of 35 to 200 mA cm^−2^, while Fe‐N_4_O/CNT maintains a high FE_CO_ (>80%) over a broad range from 35 to 400 mA cm^−2^, reaching a peak of 94.15%. It also exhibits a remarkable FE_CO_ of 86.71% at 35 mA cm^−2^ with an overpotential of 860 mV. Additionally, Fe‐N_4_O/CNT shows high FE_CO_ (>80%) across a wide current density range from 50 to 300 mA cm^−2^ in neutral electrolytes (Figure S11), and similarly high FE_CO_ (>80%) from 50 to 400 mA cm^−2^ in alkaline medium (Figure S12b), with a peak FE_CO_ of around 94% (Figure S12b). The improved activity of Fe‐N_4_O/CNT across all three environments compared to Fe‐N_4_/CNT suggests that axial oxygen ligands greatly enhance the selectivity of CO_2_RR toward CO.

Moreover, Fe‐N_4_O/CNT shows an impressive CO partial current density (*j_CO_*) of −141.22 mA cm^−2^ at −1.18 V vs. RHE, about 26.75 times higher than that of Fe‐N_4_/CNT (∼−5.28 mA cm^−2^, Figure 2c). The maximum *j_CO_* of −335.16 mA cm^−2^ achieved by Fe‐N_4_O/CNT at −1.39 V vs. RHE is suitable for industrial applications (Figure 2c). The presence of Fe NPs in the sample would further decrease the CO_2_RR activity under acidic conditions (Figure S13c). Additionally, the *j_CO_* values for Fe‐N_4_O/CNT and Fe‐N_4_/CNT were measured under neutral and alkaline conditions. Fe‐N_4_O/CNT reached high *j_CO_* values of approximately 275 and 324 mA cm^−2^ in neutral (Figure S14c) and alkaline (Figure S15c) media, respectively, which are about 42 times higher than the maximum *j_CO_* of Fe‐N_4_/CNT. As expected, Fe‐N_4_/CNT achieved a maximum CO production rate of 6.27 mmol cm^−2^ h^−1^, significantly exceeding the 0.15 mmol cm^−2^ h^−1^ rate of Fe‐N_4_/CNT (Figure 2d), showing that axial oxygen is key to boosting the activity of Fe single atoms in acidic conditions. To explore the specific activity differences between Fe‐N_4_O/CNT and Fe‐N_4_/CNT, we calculated the current density per mg of Fe site, as shown in Figure 2d. Fe‐N_4_O/CNT reached a maximum mass activity of 76.17 A mg^−1^, about 43 times greater than Fe‐N_4_/CNT (1.78 A mg^−1^, Figure 2e). This high mass activity exceeds most reported Fe single‐atom catalysts (SACs) in the literature (Figure 2f; Tables S3 and S4). [29, 30, 31, 32, 33, 34, 35, 36, 37, 38, 39]

This outstanding performance prompted us to calculate the CO turnover frequency (TOF) of Fe‐N_4_O/CNT (Figure S16), which also shows significant enhancement compared to Fe‐N_4_/CNT. The introduction of axial oxygen markedly improves CO production on Fe SACs, with Fe‐N_4_O/CNT achieving a high TOF_CO_ of ∼22 s^−1^ (79200 h^−1^), whereas Fe‐N_4_/CNT exhibited a low TOF_CO_ of 0.52 s^−1^ (1872 h^−1^). The electrochemically active surface area (ECSA) is a crucial factor influencing the intrinsic activity of catalysts. We assessed the double‐layer capacitances (*C*_dl_) using cyclic voltammetry (CV) in an unpolarized region (Figure S17). Fe‐N_4_O/CNT exhibited a *C*_dl_ of 0.211 mF cm^−2^, approximately 1.9 times lower than that of Fe‐N_4_/CNT (0.401 mF cm^−2^), potentially due to the lower Fe content in Fe‐N_4_O/CNT (Table S2). However, Fe‐N_4_O/CNT demonstrated a higher CO current density normalized by ECSA than Fe‐N_4_/CNT, as shown in Figure S18. Fe‐N_4_O/CNT achieved a maximum *j_(CO)ECSA_* of ∼63.60 mA cm^−2^, nearly 43 times that of Fe‐N_4_/CNT. This suggests that Fe‐N_4_O/CNT can provide more active Fe sites through the synergistic effect of axial oxygen, thereby enhancing catalytic activity for CO production. Meanwhile, the SCN^−^ poisoning experiment demonstrates that Fe–N_4_O is the genuine active site (Figure S19). Nyquist plots measured under open‐circuit potential (OCP) conditions showed similar radii for Fe‐N_4_O/CNT and Fe‐N_4_/CNT (Figure S20), indicating that the difference in activity is primarily due to intrinsic activity.

### 2.4 Intrinsic Activity and Mechanistic Studies for CO_2_RR Performance

**FIGURE 3:**
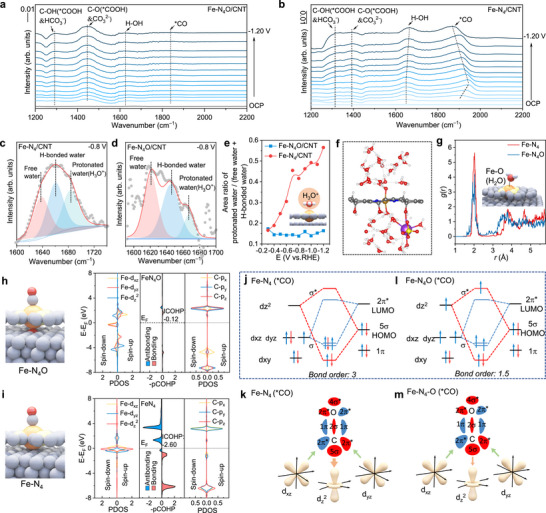
Analysis of intrinsic activity for Fe–N_4_O and Fe–N_4_ structures. (a) In situ infrared spectroscopy of Fe‐N_4_O/CNT (without iR compensation). (b) In situ infrared spectroscopy of Fe‐N_4_/CNT (without iR compensation). ATR‐SEIRAS of interfacial water on (c) Fe‐N_4_/CNT and (d) Fe‐N_4_O/CNT at −0.80 V in CO_2_‐saturated 0.5 M K_2_SO_4_ + H_2_SO_4_ (pH = 1). (e) Potential‐dependent area ratio of protonated water to (H‐bonded water + free water) on Fe‐N_4_/CNT and Fe‐N_4_O/CNT, with white indicating H, red for O, orange for Fe, grey for C, and yellow for N. (f) A typical model of Fe‐N_4_O/CNT used for MD simulations, with red representing O, white for H, blue for N, orange for Fe, purple for K^+^, yellow for S, and grey for C. (g) *g*(r) of H_2_O surrounding the Fe center for Fe‐N_4_/CNT and Fe‐N_4_O/CNT. (h) The Fe‐N_4_O model and projected crystal orbital Hamilton population (pCOHP) of Fe─C bonds, along with the corresponding projected density of states (PDOS). E‐E_f_ indicates energy relative to the Fermi level. ICOHP refers to the integrated crystal orbital Hamilton population. (i) The Fe‐N_4_ model and pCOHPs of Fe─C bonds, with the associated PDOS. E‐E_f_ denotes energy relative to the Fermi level. (j) Possible orbital interactions between Fe‐N_4_ and *CO, and (k) a schematic of σ and π‐donation bonds between CO and the 3*d* orbitals of Fe‐N_4_, where the arrow size indicates the binding strength of 3*d*_z_^2^‐5*σ* (red) and 3*d*_x_^z^/*d*_y_^z^‐2π* (green). (l) Possible orbital interactions between Fe‐N_4_O and *CO, and (m) a schematic of σ and *π*‐donation bonds between *CO and the 3*d* orbitals of Fe‐N_4_O, with arrow size representing the binding strength of 3*d*_z_^2^‐5*σ* (red) and 3*d*_x_^z^/*d*_y_^z^‐2π* (green).

To examine the notable differences in selectivity between Fe‐N_4_O/CNT and Fe‐N_4_/CNT, we used in situ attenuated total reflection surface‐enhanced infrared absorption spectroscopy (ATR‐SEIRAS) in CO_2_‐saturated 0.5 M K_2_SO_4_ (pH = 2) to gather detailed molecular vibration data at the catalyst interface (Figure 3a,b). [40] The peak at 1298 cm^−1^ is linked to the OH‐deformation of the *COOH intermediate and interfacial bicarbonate species, [41] while a prominent peak at 1430 cm^−1^ corresponds to the C─O stretching of the *COOH intermediate and interfacial carbonate [42]. These results show that the formation of the *COOH intermediate is a key step in reducing CO_2_ to CO at the Fe site, and that the formation of (bi)carbonate results from the alkaline environment at the catalyst interface. [6]

In addition to the characteristic vibrational bands associated with interfacial adsorption species such as *COOH, CO_3_^2−^, and HCO_3_^−^, a distinct shift in the water‐related band was observed for the Fe‐N_4_/CNT sample. As shown in Figure 3a,b, with increasing applied potential, the water band on Fe‐N_4_/CNT continuously blueshifts to higher wavenumbers in the infrared spectrum. In contrast, the water band position on Fe‐N_4_O/CNT remains unchanged across the same potential range. To clarify this behavior, we analyzed the bending vibration of H–O–H in the 1500–1750 cm^−1^ region, which was deconvoluted into three components: ∼1610 cm^−1^ (free water), [43, 44] ∼1640 cm^−1^ (hydrogen‐bonded water), [45, 46] and ∼1690 cm^−1^ (protonated water, H_3_O^+^), [47] as shown in Figures 3c,d and S21 and S22. Compared with Fe‐N_4_O/CNT, the area ratio of protonated water to the sum of free and H‐bonded water at the Fe‐N_4_/CNT interface increases with more negative potentials. At −1.2 V vs. RHE, the amount of protonated water at the Fe‐N_4_/CNT interface is approximately 3.5 times that of Fe‐N_4_O/CNT (Figure 3e), indicating that the unique structure of Fe‐N_4_O/CNT helps suppress H_3_O^+^ accumulation under negative electric fields.

To better understand the lower interfacial H_3_O^+^ concentration on Fe‐N_4_O/CNT, we conducted rotating disk electrode measurements to investigate the HER kinetics at the cathodic interface under acidic conditions. The results show that, compared to Fe–N_4_/CNT, H_3_O^+^ adsorption and reduction are less favorable on Fe‐N_4_O/CNT, leading to reduced HER activity (Figures S23 and S24). Consistently, we calculated the bond order between H_3_O^+^ and different Fe coordination sites [19]. The Fe–N_4_ site has a bond order of 2 with H_3_O^+^, which is significantly higher than the 0.5 value for the Fe–N_4_O site (Figure S25). Additionally, theoretical calculations further reveal that Fe‐N_4_O exhibits a weaker Fe–O interaction with H_2_O than Fe–N_4_ (Figure S26), which inhibits proton diffusion toward the Fe site through the interfacial water network. This result indicates a much weaker interaction between H_3_O^+^ and Fe‐N_4_O, contributing to its enhanced ability to suppress HER.

Meanwhile, to simulate the microenvironment surrounding the catalysts, molecular dynamics (MD) simulations were conducted (Figures 3f and S27, see Methods for details). We examined the effects of different interfacial environments and varying concentrations of charged species, including K^+^ and H^+^, on H_2_O transport. Particular attention was given to the spatial distribution of H_2_O molecules relative to the catalytic centers (Figure 3g) to assess proton transport toward the cathode interface under an applied electric field. To quantify the distribution of H_2_O species on the catalyst interface, radial distribution functions *g*(r) were calculated to analyze how close H_2_O molecules are to the catalytic centers. Specifically, the distribution of H_2_O near the central Fe atoms in Fe‐N_4_O and Fe‐N_4_ (Figure 3g) shows a clear peak at around 2.0 Å, representing the first solvation shell. Notably, the intensity of the first solvation shell around Fe‐N_4_O is lower than around Fe‐N_4_, indicating fewer interfacial H_2_O molecules on Fe‐N_4_O. The reduced number of H_2_O molecules on the catalyst surface limits rapid proton transfer to the cathode interface via the Grotthuss mechanism [40], which inhibits hydrogen evolution at high current density. This finding supports the in situ infrared data.

Additionally, a notable peak around 1945 cm^−1^, corresponding to atop‐adsorbed *CO (*CO_atop_, Figure 3b) [48], was identified on the Fe‐N_4_/CNT surface, with its signal intensity increasing as the applied potential rose. The *CO from the small peak was observed in Fe‐N_4_O/CNT (Figure 3a), indicating that *CO easily desorbs from the Fe sites of Fe‐N_4_O/CNT (Figure S28). Conversely, a significant shift in the *CO peak was seen in Fe‐N_4_/CNT, moving from 1944 cm^−1^ at −0.3 V vs. RHE to 1868 cm^−1^ at −1.2 V vs. RHE. This shift suggests that the electric field attraction makes CO desorption more difficult [49], making *CO desorption the rate‐determining step (RDS) in the electrochemical reduction of CO_2_ to CO at the Fe site of Fe‐N_4_/CNT. This is further supported by CO temperature‐programmed desorption (CO‐TPD) analysis, where the main desorption peak for Fe‐N_4_/CNT (740.77°C) appears at a higher temperature than that for Fe‐N_4_O/CNT (389.47°C, Figure S29), indicating a stronger *CO bond on Fe‐N_4_/CNT. These results align with in situ ATR‐SEIRAS data and show that adding axial oxygen changes the RDS of electrochemical CO_2_ to CO conversion on Fe single atoms, thus aiding CO_2_RR under acidic conditions.

To further investigate how spin‐state optimization facilitates the desorption of the *CO intermediate, we conducted comprehensive research using spin‐polarized DFT calculations (Figure S30). First‐principles calculations were employed to examine the impact of axial oxygen on the electron distribution at the Fe active site. We calculated the effective magnetic moments (*µ*_eff_) of 0 *µ*_B_ and 1.9 *µ*_B_ for Fe sites in Fe‐N_4_/CNT (Figure 3h) and Fe‐N_4_O/CNT (Figure 3i), respectively. This confirms that the spin state of the monodispersed Fe in the Fe‐N_4_ matrix was changed by the introduction of axial oxygen, consistent with structural analysis (Figure 1j). Based on the projected crystal orbital Hamilton population (pCOHP) analysis of Fe─C bonds between the Fe site and CO intermediate, the additional bonding/antibonding energy intensity at the Fe site and CO in Fe‐N_4_O/CNT was lower than in Fe‐N_4_/CNT, indicating weakened adsorption strength.

In contrast, low spin increased the pCOHP of the bonding and antibonding orbitals in the Fe─C bonds of Fe‐N_4_/CNT, indicating a substantial enhancement in Fe─C interaction. Compared with Fe‐N_4_‐CO (integrated crystal orbital Hamilton population (ICOHP): −2.60), the partial crystal orbital Hamilton population (pCOHP) of Fe‐N_4_O‐CO decreases and shifts positively (ICOHP: −0.12, Figure 3h), indicating that the Fe─C bonds in Fe‐N_4_O‐CO are much weaker than those in Fe‐N_4_‐CO. [50] Furthermore, the projected density of states (PDOS) of the C(CO)‐2p orbital for Fe‐N_4_‐CO (Figure 3i) shows that the partial energy levels of C‐2*p_x_* and 2*p_y_* are close to the Fermi level, indicating that the Fe‐N_4_ site has a stronger interaction with CO.

Based on the pCOHP calculation results, the interactions between CO molecular frontier orbitals (5*σ* and 2π*) and the 3*d* orbitals of Fe‐N_4_ and Fe‐N_4_O are depicted in Figure 3j–m. Compared to Fe‐N_4_ (Figure 3j), the 3*d*_z_^2^ orbital of Fe‐N_4_O is partially occupied (Figure 3l), forming a σ* orbital with *CO, with one electron filling the σ* orbital. According to orbital hybridization theory, occupation of this antibonding state weakens the Fe–CO bonding interaction, leading to weaker *CO adsorption on Fe‐N_4_O (Figure 3m) [51, 52]. This interaction is less prominent in the LS‐state Fe–N_4_ complex, which maintains stronger bonding (Figure 3k). These findings are supported by bond order (BO) calculations, where *CO on Fe–N_4_ exhibits a bond order of 3, compared to only 1.5 on Fe–N_4_O. A lower BO indicates reduced orbital overlap and weaker adsorption strength in the MS configuration. Additionally, DFT calculations of intermediate adsorption on both Fe sites show that *CO desorption is the rate‐determining step in CO_2_RR (Figures S31–S33), influencing catalytic performance under acidic conditions at high current densities. Overall, in situ characterizations and DFT calculations reveal that spin‐state modulation at Fe sites reduces their interaction with interfacial water, thereby limiting H_3_O^+^ diffusion to the active surface. Simultaneously, it decreases the binding strength to *CO intermediates, exposing active sites and enabling excellent CO_2_RR performance in acidic conditions.

### 2.5 Electrochemical Performance of Fe‐N_4_O/CNT in PEM‐PSE Reactor

**FIGURE 4:**
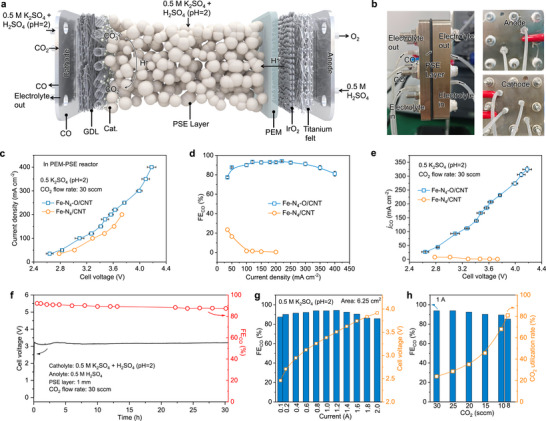
Electrochemical CO_2_RR performance of Fe‐N_4_O/CNT in a PEM‐PSE reactor. (a) Schematic illustration of electroreduction of CO_2_ to CO in our PEM‐PSE reactor. (b) Photograph of our PEM‐PSE reactor. (c) *I–V* curve of Fe‐N_4_O/CNT and Fe‐N_4_/CNT. (d) FE_CO_ of Fe‐N_4_O/CNT and Fe‐N_4_/CNT. (e) j_CO_ of Fe‐N_4_O/CNT and Fe‐N_4_/CNT. (f) Stability test for Fe‐N_4_O/CNT at 100 mA cm^−2^ in the PEM‐PSE reactor. (g) *I–V* curve and FE_CO_ of Fe‐N_4_O/CNT in our PEM‐PSE reactor with a reaction area of 6.25 cm^2^. (h) FE_CO_ and CO_2_ utilization rate (at 1 A) of Fe‐N_4_O/CNT under different CO_2_ flow rates (area: 6.25 cm^2^). The error bars in c, d, and e represent the standard deviation of three independent measurements. Data are shown as mean values ± s.d.

To further evaluate the industrial viability of the Fe–N_4_O/CNT catalyst, we integrated it into a membrane electrode assembly as the cathode and tested its performance in a full‐cell setup. Initial experiments with a self‐assembled proton exchange membrane (PEM) reactor showed poor CO_2_ reduction activity despite adding metal cations (0.5 M K_2_SO_4 _+ H_2_SO_4_ (pH = 2)) at the anode. The Faradaic efficiency for CO (FE_CO_) stayed below 50% across a current density range of 35–400 mA cm^−2^ (Figure S34). This was mainly due to the quick transport of protons through the PEM, which created an overly acidic environment at the cathode interface. To address this, we added an acidic proton‐conducting solid electrolyte (PSE) layer between the PEM and the cathode (Figures S35 and 4a). This PEM‐PSE structure lowered the full‐cell voltage and moderated local acidity at the cathode. The buffered, acidic environment provided by the PSE also helped reduce carbonate and bicarbonate formation. During operation, pure CO_2_ was continuously supplied to the cathode for electrochemical reduction to CO. Simultaneously, 0.5 M H_2_SO_4_ was circulated in the anode chamber to facilitate oxygen evolution (OER), producing protons that diffused through both the PEM and PSE to reach the cathode. These protons reacted with migrating cations at the catalyst/PSE interface, adjusting the local reaction environment to promote CO production. Additionally, carbonate (CO_3_^2−)^ and bicarbonate (HCO_3_^−)^ at the interface reacted with protons to regenerate CO_2_, minimizing salt buildup and carbon loss. The operational setup of the PEM‐PSE reactor is shown in Figure 4b.

The current–voltage (*I–V*) performance (Figure S36) of Fe‐N_4_/CNT and Fe‐N_4_O/CNT was evaluated in the PEM‐PSE reactor using 0.5 M K_2_SO_4_ + H_2_SO_4_ (pH = 2) as the circulating electrolyte in the PSE layer. As shown in Figure 4c, the cell voltage increased with current density, reaching 400 mA cm^−2^ at approximately 4.18 V. In comparison, the reactor without a PSE layer exhibited higher resistance (Figure S37), resulting in a higher cell voltage. At 3.57 V, Fe‐N_4_O/CNT achieved a current density of 200 mA cm^−2^, while Fe‐N_4_/CNT required a higher voltage of 3.73 V to reach the same level. Faradaic efficiencies for CO generation were further tested across different current densities. Fe‐N_4_/CNT showed a steady decrease in CO selectivity with increasing current, reaching a maximum FE_CO_ of 23.40%. In contrast, Fe‐N_4_O/CNT displayed a peak‐shaped trend, with a maximum FE_CO_ of 94.06% and maintaining FE_CO_ values above 80% across the entire tested range of 50 to 400 mA cm^−2^. Additionally, the *j*_CO_ reached 324.55 mA cm^−2^ (CO production rate: 6.06 mmol h^−1^ cm^−2^, Figure S38) for Fe‐N_4_O/CNT, greatly surpassing Fe‐N_4_/CNT, which only reached 3.73 mA cm^−2^ under the same conditions. These results demonstrate the potential industrial use of the Fe‐N_4_O/CNT‐based PEM‐PSE reactor for acidic CO_2_ electrolysis.

In addition to the excellent CO selectivity under acidic conditions, we further evaluated the stability of the PEM‐PSE reactor built with Fe‐N_4_O/CNT at a current density of 100 mA cm^−2^. During 30 h of continuous operation, FE_CO_ remained stable at 87.43%. Post‐reaction XPS analysis (Figure S39) confirmed the presence of Fe species, indicating the catalyst's structural stability. Meanwhile, Fe‐N_4_O/CNT also performs well in an alkaline membrane electrode system (Figures S40–S45). To assess the system's scalability, we increased the cathode area to 6.25 cm^2^, which resulted in a measured resistance of 0.49 Ω (Figure S46). A stepwise increase in current from 0.1 A to 2.0 A led to consistently high CO Faradaic efficiencies above 85%. At 2.0 A (corresponding to 320 mA cm^−2^), the cell voltage reached 3.92 V, similar to that in a 1 cm^2^ system, indicating minimal voltage rise and good scalability. To evaluate CO_2_ utilization at high current, we held the current at 1 A while varying the CO_2_ flow rate. At 8 sccm, the system achieved a CO Faradaic efficiency of 85.23%, translating to a CO_2_ utilization rate of 80.97%. These results highlight the system's high CO_2_ conversion efficiency and its potential for commercial application.

### 2.6 Performance Improvements and Techno‐Economic Analysis

**FIGURE 5:**
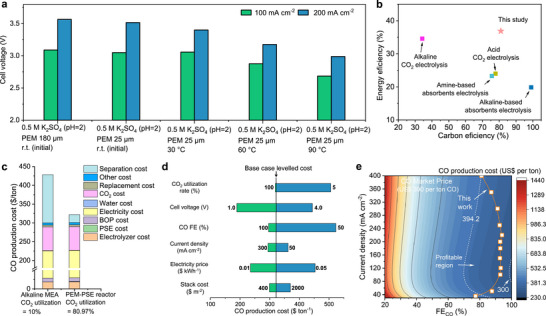
CO_2_RR enhancements in the PEM‐PSE reactor. (a) Strategies to further reduce energy consumption: cell voltages (at 100 mA cm^−2^ and 200 mA cm^−2^) in PSE reactors with different PEM thicknesses (180 µm vs. 25 µm), across various operation temperatures (30, 60, and 90°C). (b) The carbon and energy efficiencies of our system compared to other CO_2_ electroreduction systems. (c) Comparison of CO production costs under alkaline and acidic conditions with various CO_2_ utilization rates. (d) Univariate sensitivity analysis of key parameters affecting CO production cost. The baseline values include a CO_2_ utilization rate of 80.97%, an electrolyzer voltage of 3.09 V, a current density of 100 mA cm^−2^, a CO Faradaic efficiency (FE) of 90%, an electricity price of $0.03 kWh^−1^, and a stack cost of $919.7m^−2^. Each parameter is varied individually to evaluate its effect on CO production costs. (e) A contour map showing the total CO production cost for acidic CO_2_ electroreduction in the PEM‐PSE reactor, based on different current densities and FE_CO_.

While our PEM–PSE reactor using Fe–N_4_O/CNT as the cathode for acidic CO_2_ reduction showed a promising carbon utilization rate, we highlight that the electrochemical performance has not yet been fully optimized. There are many opportunities to further improve reactor efficiency, including using more conductive PEMs, enhancing the ionic conductivity of solid electrolyte particles, and operating at higher temperatures. To demonstrate the potential for performance improvements, we performed additional experiments on key factors that significantly boost energy efficiency, especially at high current densities (Figures 5a and S47). Although these reductions in energy use are promising, continued optimization of the reactor design and process parameters will be essential to move toward practical application.

Based on the electrochemical performance of acidic CO_2_ reduction in the PEM–PSE reactor, we evaluated both carbon utilization and energy efficiency to assess technological progress. As shown in Figure 5b, we compare various reported CO_2_ electrolysis systems, including alkaline and acidic electrolysis, as well as CO_2_ capture liquid electrolysis, and find that our PEM–PSE reactor, employing Fe–N_4_O/CNT as the cathode, delivers significantly higher carbon and energy efficiencies, reaching 80.97% and 36.89%, respectively [53, 54, 55, 56, 57].

Based on the electrochemical performance of our PEM–PSE reactor system for acidic CO_2_ reduction, we conducted a techno‐economic analysis (TEA) to evaluate its economic viability and scalability, following established methodologies reported in previous studies [58]. As shown in Figure 5c, when the CO_2_ utilization in alkaline systems is as low as 10%, the overall CO production cost rises significantly (Figure 5c). This is primarily due to high capital expenses for building large‐scale gas separation units and increased electricity requirements for downstream gas purification, resulting in a cost exceeding $400 per tonne of CO (specifically, $428.26 per tonne). In contrast, with our PEM–PSE reactor design, the high CO_2_ utilization allows for a substantial reduction in separation‐related costs, bringing the production cost down to $321.87 per tonne of CO. These findings highlight the importance of improving CO_2_ utilization to lower the overall cost of CO production.

A more detailed global sensitivity analysis (Figure 5d) further shows that the cost of CO production is highly responsive to changes in electricity price (ranging from US$0.01 to US$0.05 per kWh), cell voltage (from 1.0 to 4.0 V), and CO_2_ utilization (from 100% to 5%). According to the TEA calculations in Supporting Note S1, the electricity price directly influences operating costs, while cell voltage affects the electricity consumption. CO_2_ utilization, on the other hand, is closely linked to the capital and operational costs of the downstream separation units needed to produce one tonne of CO. Therefore, targeted strategies are necessary to reduce these risks, such as avoiding increases in cell voltage or decreases in conversion efficiency at certain current densities. Additionally, tracking changes in energy policy and grid electricity prices will be crucial for maintaining the economic viability of acidic CO_2_ electrolysis.

To better understand cost‐reduction options, we plotted the relationship between CO production cost and electrolyzer performance (Figures 5e and S48). The analysis shows that, at current performance levels, the CO production cost stays above $300 per tonne across a broad range of current densities (35–40 mA cm^−2^). Fortunately, under the current U.S. carbon policy (45Q policy), [59] converting 1 tonne of CO_2_ qualifies for a $60 tax credit. Since producing 1 tonne of CO consumes about 1.57 tonnes of CO_2_, this effectively equals about $94.2 in credits per tonne of CO. Under these conditions, our system allows for economically viable CO production. Further reductions in electricity price would boost profitability even more (Figure S49). These results highlight the near‐term commercial potential of the proposed acidic CO_2_ electrolysis system and its ability to efficiently utilize carbon resources.

## 3 Conclusion

In conclusion, we introduce an axial oxygen coordination strategy to adjust the spin state of Fe‐N‐C catalytic sites, enabling a transition from a low‐spin (LS, t_2g_^5^ e_g_^0^) to a medium‐spin (MS, t_2g_^4^ e_g_^1^) state. The resulting Fe–N_4_O sites deliver a peak mass activity of 76.17 A mg_Fe_^−1^ and a CO partial current density of 335 mA cm^−2^ under acidic conditions, significantly surpassing the benchmark Fe–N_4_ catalyst (∼7.9 mA cm^−2^). Combined experimental and theoretical analyses show that the axial oxygen ligand influences the electronic environment of the Fe center, promoting a spin‐state transition by increasing the separation of d‐orbital electrons. This spin reconfiguration weakens the interaction between Fe sites and H_3_O^+^, while also reducing the desorption energy of *CO, facilitating efficient CO_2_ electroreduction in acidic media. Thanks to the Fe–N_4_O structure, the system achieves a CO partial current density of 324.55 mA cm^−2^ and a CO_2_ utilization efficiency of 80.97% in a PEM–PSE reactor, demonstrating promising potential for scalable CO_2_ conversion powered by renewable electricity.

## Author Contributions

Y. Y., C. W., X. Z., and S. P. L. conceived the project and designed the experiments. S. G., X. Z., and S. P. L. conceived the idea. Y. Y. performed the theoretical study. S. G., Y. Z., Q. X., X. H., W. L., and Y. Y. Z., carried out the electrochemical study. X. S. and Z. W. performed the product gas detection. S. G., J. W., H. X., and X. L. performed catalyst characterization. S. G., X. Z., and S. P. L. wrote the manuscript with support from all authors.

## Conflicts of Interest

The authors declare no conflicts of interest.

## Supporting information




**Supporting File 1**: anie72079‐sup‐0001‐SuppMat.docx.

## Data Availability

The data that supports the findings of this study are available in the supporting information of this article.
