# Supplementary material for: Spin‐State Modulation of Atomic Iron Sites Enables Efficient CO2 Electroreduction in Acid Medium
Source: Angew Chem Int Ed Engl. 2026 Apr 5;65(20):e9239759. doi: 10.1002/anie.9239759 (PMC13159417; doi:10.1002/anie.9239759)
Supplement: Supplementary file 1 — Supporting File 1: anie72079‐sup‐0001‐SuppMat.docx. [file ANIE-65-e9239759-s001.docx]

Supporting Information

Spin-State Modulation of Atomic Iron Sites Enables Efficient CO_2_ Electroreduction in Acid Medium

Shanhe Gong^1,2,3,4,5+^, Yanjie Zhai^2+^, Qing Xia^2+^, Xu Han^4^, Weisong Li^2^, Yiran Ying^6^*, Jie Wu^2^, Yingying Zhou^2^, Xiaojie She^1,3^, Zhaolong Wang^1,3^, Chundu Wu^5^*, Xiaomeng Lv^4^, Xiao Zhang^2,7^*, and Shu Ping Lau^1^^,3^*

^1^Department of Applied Physics, The Hong Kong Polytechnic University, Hong Kong SAR.

^2^Department of Mechanical Engineering, The Hong Kong Polytechnic University, Hong Kong SAR.

^3^Photonics Research Institute, The Hong Kong Polytechnic University, Hong Kong SAR.

^4^School of Chemistry and Chemical Engineering, Jiangsu University, Zhenjiang, P. R. China.

^5^School of Agricultural Engineering, Jiangsu University, Zhenjiang, P. R. China.

^6^State Key Laboratory of Solidification Processing, Center for Nano Energy Materials, Northwestern Polytechnical University and Shaanxi Joint Laboratory of Graphene (NPU), Xian, P. R. China

^7^Shenzhen Research Institute, The Hong Kong Polytechnic University, Shenzhen, Guangdong, P. R. China

^+^These authors contribute equally.

^*^Corresponding authors:

Yiran Ying, Chundu Wu, Xiao Zhang and Shu Ping Lau

*E-mail:*

Yiran Ying: yiranying@nwpu.edu.cn

Chundu Wu: wucd@ujs.edu.cn

Xiao Zhang: [xiao1.zhang@polyu.edu.hk](mailto:xiao1.zhang@polyu.edu.hk)

Shu Ping Lau: [apsplau@polyu.edu.hk](mailto:apsplau@polyu.edu.hk)

**1. Supplementary Experimental Section**

**1.1 Chemical agent**

Ethanol (CH_3_CH_2_OH, ≥99.7%), ferric trichloride (≥98%), 2-propanol ((CH_3_)_2_CHOH, ≥99.0%), 1,8-diazabicyclo[5.4.0]undec-7-ene (DBU, ≥98.0%), N,N-dimethylformamide (DMF, ≥99.9%), potassium hydroxide (KOH, ≥85.0%), hydrochloric acid (HCl, 36.0%–38.0%), potassium bicarbonate (KHCO_3_, ≥99.9%), sulfuric acid (H_2_SO_4_, ≥99.8%), and potassium sulfate (K_2_SO_4_, ≥99.0%) were all purchased from Sinopharm Chemical Reagent Co., Ltd. Hydroxyl-functionalized carbon nanotube (CNT-OH, ≥95%), graphitized carbon nanotube (CNT, ≥99.9%), and triethylamine (≥99.5%) were purchased from Macklin Chemical Company. Protoporphyrin IX iron (III) chloride (FePPCl, ≥95%) was purchased from Aladdin Chemical Company. Dowex 50W X8-based porous solid-state electrolyte (PSE, 200-400 mesh, H^+^) was purchased from Sigma-Aldrich. 1,2-dicyanobenzene (≥95.0%) was purchased from Bidepharm Chemical Company. High-purity carbon dioxide gas (CO_2_, 99.999%) and high-purity argon (Ar, 99.999%) were purchased from Jiangsu SoPo Gas Co., Ltd. (China). Millipore water (18.2 MΩ·cm) was used throughout all experiments.

**1.2 Preparation of catalyst**

**1.2.1 Synthesis of Fe-N_4_O/CNT catalyst**

To synthesize Fe–N_4_O/CNT, 100 mg of hydroxyl-functionalized multi-walled carbon nanotubes (CNT–OH) were dispersed in 50 mL of ethanol and sonicated for 30 minutes. Subsequently, 5 mg of protoporphyrin IX iron (III) chloride (FePPCl) was added to the suspension and subjected to 30 minutes of ultrasonication. Then, 0.5 mL of triethylamine was introduced, and the mixture was refluxed at 90 °C for 48 hours. The product was collected via vacuum filtration, thoroughly washed with ethanol and deionized water to remove residual impurities, and vacuum-dried. The obtained FeTPP@CNT was then heat-treated under an argon atmosphere at 800 °C for 2 hours to produce the final Fe–N_4_O/CNT catalyst. The amount of FePPCl was increased to 25 mg, and the sample Fe–N_4_O/CNT-5 was prepared using a similar method as described above.

**1.2.2 Synthesis of Fe-N_4_/CNT catalyst**

To synthesize Fe–N_4_/CNT, iron(III) phthalocyanine (Fe(III)Pc) was first prepared using a solvothermal method. Specifically, 32 mg of ferric trichloride and 180 mg of 1,2-dicyanobenzene were added to 50 mL of ethanol and ultrasonicated for 1 hour to form a uniform suspension. Then, 0.5 mL of 1,8-diazabicyclo[5.4.0]undec-7-ene (DBU) was added as a catalyst. The mixture was transferred to a 100 mL Teflon-lined stainless-steel autoclave and heated at 180 °C for 8 hours. During this process, strong bases formed in situ nucleophilically attack the cyano groups of phthalonitrile, promoting the formation of metal phthalocyanines. The resulting precipitate was collected by vacuum filtration and washed sequentially with 0.5 M HCl, 0.5 M KOH, deionized water, and methanol to obtain Fe(III)Pc. Next, 5 mg of commercial FePc was dispersed in 50 mL of N,N-dimethylformamide (DMF) and ultrasonicated for 30 minutes to produce solution A. Meanwhile, 100 mg of graphitized CNTs were dispersed in 100 mL of DMF and ultrasonicated for 30 minutes to produce solution B. Solution A was then gradually added to solution B under vigorous stirring, and the mixture was stirred continuously for 24 hours. The product was collected by vacuum filtration and washed repeatedly with DMF and ethanol until the filtrate was colorless. The final FePc/CNT composite was vacuum-dried and heat-treated under an argon atmosphere at 800 °C for 2 hours to produce the Fe–N_4_/CNT catalyst.

**1.3. Electrochemical measurements**

**1.3.1 Flow cell test:** To prepare the catalyst ink, 9 mg of the sample was dispersed in 2 mL of 1-pentanol and 60 μL of Nafion solution (5 wt%) and sonicated for 1 hour to create a homogeneous suspension. The resulting ink was uniformly air-brushed onto a 3 × 3 cm^2^ piece of hydrophobic carbon paper (Sigracet 28 BC gas diffusion layer) to achieve a catalyst loading of 1 mg/cm^2^, followed by drying at 60 °C overnight. The Ag/AgCl electrode (neutral, alkaline) and the Hg/Hg_2_SO_4_ electrode (acidic) served as the reference electrodes, while a platinum foil (geometric area: 1 cm^2^) was used as the counter electrode. All measured potentials were referenced to the reversible hydrogen electrode (RHE) using the Nernst equation at 25 °C, as follows (equation S1and equation S2).

$E \left( vs.\mathrm{RHE} \right)=E \left( vs.Ag/AgCl \right)+0.2046 V+0.059\times pH-80\%iRu$ (S1)

$E \left( vs.\mathrm{RHE} \right)=E \left( vs.Hg/\mathrm{Hg}_{2}SO_{4} \right)+0.64 V+0.059\times pH-80\%iRu$ (S2)

where i represents the current and Ru is the solution resistance. Electrochemical measurements were performed using a CHI 1140C workstation (Shanghai, CHI760e). Gaseous products were analyzed by online gas chromatography (GC-2030, Shimadzu) equipped with both a thermal conductivity detector (TCD) and a flame ionization detector (FID). Liquid-phase products were quantified via proton nuclear magnetic resonance (^1^H NMR) spectroscopy, using DMSO as the internal standard. Before electrolysis, each cathode was activated by cyclic voltammetry for 20 minutes. In alkaline and neutral conditions, 1 M KOH and 1 M KHCO_3_ were used as the catholyte and anolyte, respectively. Under acidic conditions, 0.5 M K_2_SO_4_ (pH = 2) served as the catholyte and 0.5 M H_2_SO_4_ as the anolyte. All electrolytes were purged with argon for 30 minutes before use. During electrolysis, electrolytes were circulated through the cathodic and anodic chambers at flow rates of 10 mL min^-1^ and 40 mL min^-1^, respectively, using a syringe pump. For flow-cell experiments, the applied potential was corrected for 80% of the measured ohmic resistance, determined via electrochemical impedance spectroscopy (EIS) under open-circuit conditions. The inlet CO_2_ flow rate into the gas chamber was controlled at 30 mL min^-1^ using a 100 sccm mass flow controller (MFC), and the outlet flow rate was monitored with a soap-film flowmeter.

Electrochemically active surface area (ECSA) and EIS measurements were conducted in an H-type cell using a Bio-Logic VMP3e electrochemical workstation. Both cathodic and anodic chambers contained 0.5 M KHCO_3_ electrolyte, with the catholyte saturated with CO_2_. ECSA was determined at a potential where Faradaic efficiency for CO_2_ reduction was negligible and was estimated from the double-layer capacitance (*C_dl_*), which is linearly related to ECSA. The catalyst loading was set at 1 mg/cm^2^. EIS measurements were performed at −0.60 V vs. RHE.

**1.3.2 Membrane electrode assembly electrolytic cell test**

Electrochemical measurements were carried out using a Bio-Logic VMP3e workstation and a CHI760e electrochemical analyzer. A 0.25 mm-thick polytetrafluoroethylene (PTFE) gasket with a 1.0 cm^2^ window defined the cathode area. The cathode consisted of an Fe–N_4_O/CNT catalyst (loading: 1 mg cm^-2^) coated onto a 1 cm^2^ gas diffusion layer (GDL), while a pre-fabricated Ir/RuO_x_/Ti mesh served as the anode. The electrolyte on the anode side was either 1 M KOH or 1 M KHCO_3_, circulated at a flow rate of 10 mL min^-1^ using a syringe pump. The cathode and anode chambers were separated by a Sustainion anion exchange membrane (AEM). CO₂ gas was supplied to the cathode at a flow rate of 30 mL min-1 via a 100 sccm mass flow controller (MFC). The CO_2_ flow rate was maintained at 30 mL min^-1^, regulated by the same MFC system. During long-term stability tests, the anolyte was replaced with 0.2 M KOH.

**1.3.3 Acid porous solid electrolyte reactor test**

Electrochemical measurements were conducted using a Bio-Logic VMP3e and a CHI 660e workstation equipped with a CHI 680C current booster. The cathodic compartment consisted of a stainless steel chamber with a serpentine flow field and four ports: two for CO_2_ gas circulation and two for electrolyte flow through a 1 mm-thick middle chamber. A 0.5 mm silicone gasket with a 1.0 cm^2^ window was used to ensure tight contact between the cathode and the porous solid electrolyte (PSE), which was filled in the 1 mm-thick middle chamber. The cathode received 30 mL min^-1^ of CO_2_ (controlled by a 100 sccm Alicat MFC). IrO_2_/Ti mesh served as the anode, separated from the solid electrolyte layer by a Nafion HP membrane (20 µm). The anode was supplied with 0.5 M H_2_SO_4_ at 2.0 mL min^-1^, while the catholyte consisted of 0.5 M K_2_SO_4_ (pH = 2).

**1.4 *In situ* electrochemical ATR-SEIRAS measurements**

*In situ* electrochemical attenuated total reflection surface-enhanced infrared absorption spectroscopy (ATR-SEIRAS) measurements were performed using a Pike Veemax III ATR electrochemical cell, equipped with a single-reflection silicon crystal coated with a gold (Au) film, operating in internal reflection mode. The spectrum was recorded on a Thermo Fisher Nicolet™ iS50 spectrometer (U.S.). All experiments were conducted in a three-electrode setup, with Fe–N_4_O/CNT as the working electrode, Ag/AgCl as the reference electrode, and platinum felt as the counter electrode. Before measurement, the 0.5 M K_2_SO_4_ electrolyte (pH = 2) was saturated with CO_2_ gas for 30 minutes. The applied potential was scanned from open-circuit potential (OCP) to −1.2 V versus the reversible hydrogen electrode (RHE).

**1.5 Evaluation of Faraday efficiency**. The Faradic efficiency of the gas product was calculated by the following equation (equation S3):

$\mathrm{FE}_{x}=\frac{n_{x}\times F(C/mol)\times V(ml/min)\times{10}^{-6}(m^{3}/ml)\times v\left( vol\% \right)\times1.013\times{10}^{5}(N/m^{2})}{8.314(N\cdot m/mol\cdot K)\times T(K)\times I_{total}(C/s)\times60(s/min)}$ (S3)

$n_{x}$: the number of electrons required to produce a product molecule (H_2_, CO = 2);

$F$: the Faradaic constant (96485 C mol^−1^);

$V$: the flow rate of CO_2_ bubbling;

$v$: the volume ratio of gas product calculated from the peak area of GC current signal;

$T$: 298.15 K;

$I_{total}$: the steady-state cell current (C/s).

**1.6 Evaluation of partial current density.** The partial current density of the gas product was calculated by the following formula (equation S4):

$j_{x}={FE}_{x}\times j_{total}$ (S4)

$j_{x}$: the partial current density (mA cm^−2^) of gas product;

${FE}_{x}$: the Faradic efficiency of gas product;

$j_{total}$: the total current density (mA cm^−2^).

**1.7 Evaluation of TOF.** The turnover frequency (TOF, s^−1^) value of gas product was calculated by the following equation (equation S5):

${TOF}_{x}=\frac{{FE}_{x}\times j_{total}}{n_{x}\times F\times\omega\times m_{cat}/M_{Fe}}$ (S5)

$\omega$: the content of iron (Fe) in the hybrid catalysts;

$m_{cat}$: the amount of the tested catalyst;

$M_{ni}$: the relative molecular weight of Fe.

**1.8 The half-cell and full-cell energy efficiencies** **(EE_half cell_ and EE_full cell_)**. The half-cell and full-cell energy efficiencies were calculated using the following equations (equation S6 and equation S7):

${EE}_{half cell}\left( \% \right)=\frac{\left( E_{OER}^{\theta}-E_{X}^{\theta} \right)\times FE}{E_{OER}^{\theta}-E_{C}}\times100\%$ (S6)

${EE}_{Full cell}\left( \% \right)=\frac{\left( E_{OER}^{\theta}-E_{X}^{\theta} \right)\times FE}{E_{full cell}}\times100\%$ (S7)

where $E_{OER}^{\theta}$ and $E_{X}^{\theta}$ are the thermodynamic potentials (versus RHE) for OER and the ECO_2_R to product x, respectively, FE is the FE of product x, ${EE}_{half cell}$ is the potential applied at the cathode and ${EE}_{Full cell}$is the cell voltage of the MEA or PEM-PSE system.

**1.9 The SPCE of CO_2_ towards producing CO was calculated as follows at 25 °C, 1 atm** (equation S8):

$\mathrm{SPCE}=\frac{J_{CO}\left( mA \right)\times60 \left( s \right)\times24.05 (L {min}^{-1})}{N\times F\times v}\times100\%$ (S8)

Where $J_{CO}$ represents the partial current of CO, and N stands for electron transfer with 2 indicating the CO product. $v$ is the outlet gas flow rate at the GC outlet, measured using a mass flow controller (MFC). The CO_2_ flow rate at the inlet was controlled to a constant value using a second mass flow controller (MFC). The GC standard curve was recalibrated using a standard gas mixture with a higher concentration (tens of thousands of parts per million).

**1.10** **Rotating disk electrode experiments:** The diffusion limiting current of the reduction of hydronium ions was calculated according to Levich equation (equation S9 and equation S10):

$i_{plateau}=0.62nFAD^{\frac{2}{3}}{v^{-}}^{\frac{1}{6}}c_{O,H^{+}}\omega^{\frac{1}{2}}$ (S9)

$S=0.62nFAD^{\frac{2}{3}}{v^{-}}^{\frac{1}{6}}c_{O,H^{+}}$ (S10)

where n is the number of electrons transfer in the reaction, $F$ is the Faraday constant (9.65×10^4^ C mol^-1^), A is the electrode surface area, $D$ is the diffusion coefficient of hydronium ions, $v$ is the kinematic viscosity of electrolyte, $c_{O,H^{+}}$ is the bulk concentration of hydronium ions, and 𝜔 is the rotating speed of the RDE (unit: rad s^-1^), $i_{plateau}$ is the current. $S$ is the linear fitting slope.

The kinetic current density can be obtained from the Koutecky-Levich equation (equation S11):

$\frac{1}{J_{tot}}=\frac{1}{J_{k}}+\frac{1}{J_{plateau}}$ (S11)

where$j_{tot}$ is the total current density, $j_{k}$ is the kinetic current density and $j_{plateau}$ is the diffusion limiting current density.

**1.11 Material Characterizations:** The morphologies of as-prepared samples were studied using TEM (Japan, JEOL-2100F), HRTEM (Japan, JEOL-2100F), HAADF-STEM (FEI Titan) with energy dispersive X-ray spectroscopy (EDX), and SEM with a field-emission scanning electron microanalyzer (Hitachi S-4800 II, Japan). Furthermore, the atomic structure of Fe-N_4_O/CNT was characterized using an FEI Themis Z transmission electron microscope operated at 200 kV and equipped with double spherical aberration (Cs) correctors. The physical structure of the samples was analyzed via X-ray diffractometry (XRD, Bruker AXS, Germany) with monochromized Cu Kα radiation (λ=1.54178 Å), and X-ray photoelectron spectroscopy (XPS) with an ESCA PHI500 spectrometer. The Fe loading of the catalysts was quantified by inductively coupled plasma mass spectrometry (ICP-MS; Agilent 7800). For ZFC measurements, samples were exposed to temperatures ranging from 2 to 300 K at a rate of 5 K/min, with an external field of 1000 Oe applied. Mössbauer spectra were recorded at room temperature in transmission mode using a proportional counter on the WSS-10 Mössbauer instrument. A ^57^Co (Rh) radioactive source provided the γ-rays, and isomer shifts were calibrated with α-Fe foil. Spectra were fitted using the least-square method based on the Lorentzian function with MossWinn 4.0 software. Electron paramagnetic resonance (EPR) spectra were captured with a Bruker EMX PLUS (Bruker Corporation) spectrometer at room temperature. The hysteresis loop was measured using LakeShore (U.S.) equipment at room temperature.

**1.12 X-ray Absorption Spectroscopy (XAS):** The sample was analyzed using XAS at the Shanghai synchrotron radiation facility in China. The spectra were processed and analyzed with the software Athena [1-2].

**1.13 Computational Details:**

All spin-polarized density functional theory (DFT) calculations were performed using the VASP code with the projector-augmented wave method [3-4]. The Perdew-Burke-Ernzerhof scheme of the generalized gradient approximation was applied to describe the exchange-correlation functional [5]. Van der Waals corrections were included using Grimme’s DFT-D3 method [6]. Energy and force convergence criteria were set at 10^-5^ eV and 0.01 eV/Å, respectively. 3×3×1 k-point meshes were used to sample the first Brillouin zone. Vacuum layers exceeding 15 Å in thickness were added to prevent spurious interactions between adjacent layers. The charge transfer was estimated through Bader charge analysis [7]. Crystal orbital Hamilton population was calculated with the LOBSTER package [8].

The computational hydrogen electrode (CHE) model was used in the free energy calculations [9], and Gibbs free energy change ΔG was computed as ΔG = ΔE + ΔZPE – TΔS + ΔH_0→T_ + ΔG_pH_, where ΔE, ΔZPE, TΔS, and ΔH_0→T_ represent changes in DFT-calculated energy, zero-point energy, entropy contribution (T = 298 K), and reaction enthalpy from zero to finite temperature T, respectively. ΔG_pH_ = k_B_Tln10×pH is the pH correction to ΔG. The solvation effect was incorporated using the implicit solvation model in the VASPsol package [10], with the relative permittivity set to 80. Ab initio molecular dynamics (AIMD) simulations were performed in the NVT ensemble (300 K) with the Nosé-Hoover thermostat [11-12], using a time step of 0.5 fs. Periodic boundary conditions were applied in all simulations. Both Fe-N_4_O and Fe-N_4_ models were derived from DFT calculations. The simulation box included one explicit H_3_O^+^ and 34 H_2_O molecules to model the local acidic environment, along with one K^+^ and one SO_4_^2-^ to ensure charge neutrality.

**Supplementary Note 1. Techno-economic analysis**

A techno-economic analysis was developed and conducted based on previously reported models [13-14]. We reported electrochemical CO_2_ reduction with a current density of 100 mA cm^–2^ and a FE_CO_ of 90%, FE_H2_ of 9.41%. Assuming a production rate of 100,000 kg per day for CO production with 350 operational days per year at the cathode.

The partial current needed is:

$$100,000\frac{kg}{day}*\frac{day}{86400s}*1,000\frac{g}{kg}*\frac{mol}{28}*{2e}^{-}*96,485\frac{c}{mol}=7976603.836A$$

The total current needed is then given by diving by the faradaic efficiency:

$$Total current=\frac{7976603.836A}{0.9}=8862893.151A$$

The electrolyzer area needed is:

$$Total electrolyzer area=\frac{8862893.151 A}{0.10\frac{A}{{cm}^{2}}}*\frac{m^{2}}{{10}^{4}{cm}^{2}}=8862.89 m^{2}$$

The power needed is given from P=UI, the cell voltage is 3.09 V for 100 mA cm^–2^:

$${Power}_{need}=3.09 V*8862893.151 A*\frac{W}{{10}^{6}MW}=27.38634 MW$$

CO_2_ flow rate needed is:

$${CO}_{2} flow rate needed=total current*0.9*\frac{86400s}{day}*\frac{1}{2\frac{e^{-}}{mol}*96485\frac{C}{mol}}*0.044\frac{kg}{mol}=157146.52 \frac{kg}{day}$$

Based on our tests, the single-pass conversion is 80.97%, the CO_2_ inlet flow rate can be defined as:

$${CO}_{2} inlet flow rate=157146.52\frac{kg}{day}*\frac{1}{0.8097}=194065.5276\frac{kg}{day}$$

The outlet gas flow rate at the cathode side is a sum of CO_2_, CO, and H_2_:

$${CO}_{2} outlet flow rate=194065.5276\frac{kg}{day}*(1-0.8097)*\frac{m^{3}}{1.98kg}*\frac{day}{24hr}=777.1606\frac{m^{3}}{hr}$$

$$CO outlet flow rate=8862893.151*0.9*\frac{1}{2e^{-}}*\frac{1}{96485\frac{C}{mol}}*0.0281\frac{kg}{mol}*\frac{m^{3}}{1.14kg}*\frac{3600s}{hr}=3668.024227\frac{m^{3}}{hr}$$

$$H_{2} outlet flow rate=8862893.151*0.0941*\frac{1}{2e^{-}}*\frac{1}{96485\frac{C}{mol}}*0.002\frac{kg}{mol}*\frac{m^{3}}{0.0899kg}*\frac{3600s}{hr}=346.14\frac{m^{3}}{hr}$$

$$Total gas outlet flow rate=777.1606\frac{m^{3}}{hr}+3668.024227\frac{m^{3}}{hr}+346.14\frac{m^{3}}{hr}=4791.324827\frac{m^{3}}{hr}$$

The water flow rate for the anodic OER reaction is

$$H_{2}O flow rate=8862893.151*\frac{1}{4e^{-}}*\frac{1}{96485\frac{C}{mol}}*0.018\frac{kg}{mol}*\frac{0.2642gal}{kg}*\frac{86400s}{day}=9435.71429\frac{gal}{day}$$

$$O2 outlet flow rate=8862893.151*\frac{1}{4e^{-}}*\frac{1}{96485\frac{C}{mol}}*0.032\frac{kg}{mol}*\frac{m^{3}}{1.429kg}*\frac{3600s}{hr}=1851.29646\frac{m^{3}}{hr}$$

**Capital costs**

The stack cost of $250.25 kW was employed according to DOE H2A analysis for central grid electrolysis. The reference electrolyzer operates at 1.75 V and 0.175 A cm^-2^ and the installation factor is 1.2. Thus, the cost per area for the reference electrolyzer is:

$$Ref.electrolyzer cost=\frac{\$250.25}{kW}*\frac{0.175A}{{cm}^{2}}*1.75V*\frac{{10}^{4}{cm}^{2}}{m^{2}}*\frac{kW}{1,000W}*1.2=\frac{\$919.7}{m^{2}}$$

Then, the electrolyzer capital cost is given by multiplying the total area:

$$Electrolyzer cost=8862.89 m^{2}*\frac{\$919.7}{m^{2}}=\$8149046.333$$

Herein, the capital recovery factor (CRF) is based on a discount rate (denoted i; we use 5% for all the CRF calculations) and the material lifetime.

$${CRF}_{Electrolyzer} =\frac{i{(1+i)}^{year}}{{(1+i)}^{year}-1}= \frac{0.05*{1.05}^{20}}{{1.05}^{20}-1}=0.0802$$

Electrolyzer cost for day

$$Electrolyzer cost=\$8149046.333*\frac{0.0802}{350}=\frac{\$1867.13388}{day}$$

**Balance of plant cost**

According to the H2A analysis [13], the balance of plant (BoP) capital cost is 35% of the total cost, while the stack is 65%:

$$BoP capital cost=\$1867.13388*0.35/0.65=\frac{\$1005.38}{day}$$

The total capital cost of pressure swing adsorption (PSA) is calculated by scaling the reference cost to the total flow rate:

$$PSA capital cost = \$1,989,043*\left( \left( \frac{4791.324827 \frac{m^{3}}{hr}}{\frac{{1000m}^{3}}{hr}} \right)^{0.7} \right)=\$5956,115.38$$

By assuming the PSA facility lifetime is the same as the electrolyzer lifetime (20 year), the PSA capital cost of the corresponding quantity of CO_2_, CO and H_2_ for day is written:

$$PSA capital cost = \$5956,115.38*\frac{0.0802}{350}=\frac{\$1364.8013}{day}$$

**Operating costs**

The price of electricity is 0.03$/kWh, the electricity cost can be defined as:

$$Electricity cost=27.38634 MW*\frac{1,000kW}{MW}*24hr*\frac{\$0.03}{kWh}=\frac{\$19718.16480}{day}$$

The PSA costs are calculated by scaling the reference costs to the flow

rates:

$$PSA=\frac{0.25kWh}{m^{3}}*4791.324827\frac{m^{3}}{hr}*\frac{\$0.03}{kWh}*\frac{24hr}{day}=\frac{\$862.438}{day}$$

The cost of the water:

$$Water cost=\left( 9435.71429\frac{gal}{day} \right)*\frac{\$0.0054}{gal}=\frac{\$50.95286}{day}$$

The cost of CO_2_ ($40/ton):

$${CO}_{2} cost=157142.8571\frac{kg}{day}*\frac{1ton}{1000 kg}*\frac{\$40}{1ton}=\frac{\$6285.714286}{day}$$

Cell compartment replacement (every 7 years)

$$Replacement cost=\$8149046.333*0.15*\frac{1year}{350day}*\frac{1}{7year}=\frac{\$498.29328}{day}$$

**Other costs**

Other costs, such as labor and maintenance, are assumed to be 2.5% of the electrolyzer cost:

$$Other cost=\$8149046.333*\frac{0.025}{350 day}=\frac{\$582.07474}{day}$$

Thus, the overall cost to electrochemical CO_2_ reduction to CO (100,000 kg/day) is

Overall cost ≈$32187.80.

**2. Supplementary Figure**


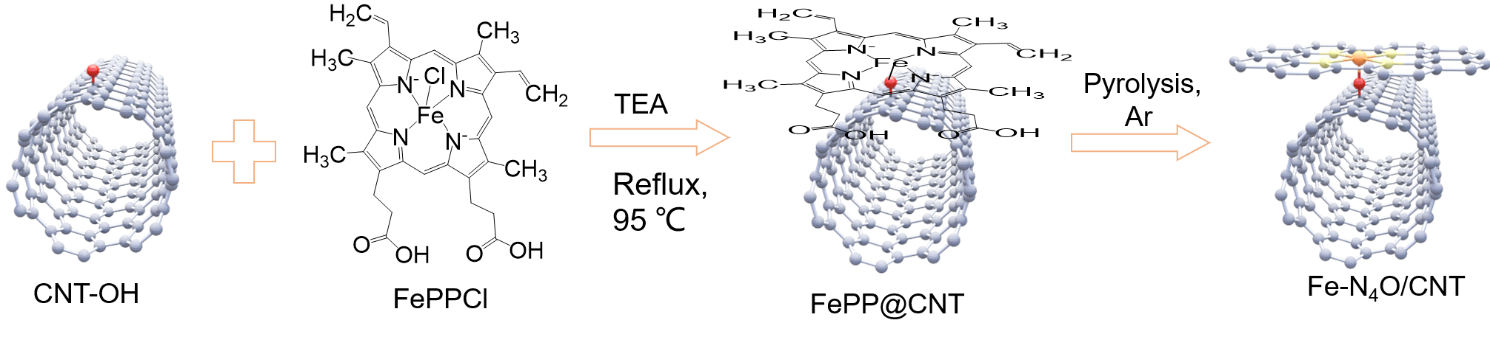


**Figure S1.** The scheme for the synthesis of Fe-N_4_O/CNT. Commercial hydroxylated CNT (CNT–OH) serves as the support, enabling axial coordination of a Fe–porphyrin precursor through a Fe–O bond to create a Fe–porphyrin/CNT intermediate. Subsequent pyrolysis transforms the molecular precursor into atomically dispersed Fe–N_4_O sites anchored on CNT.


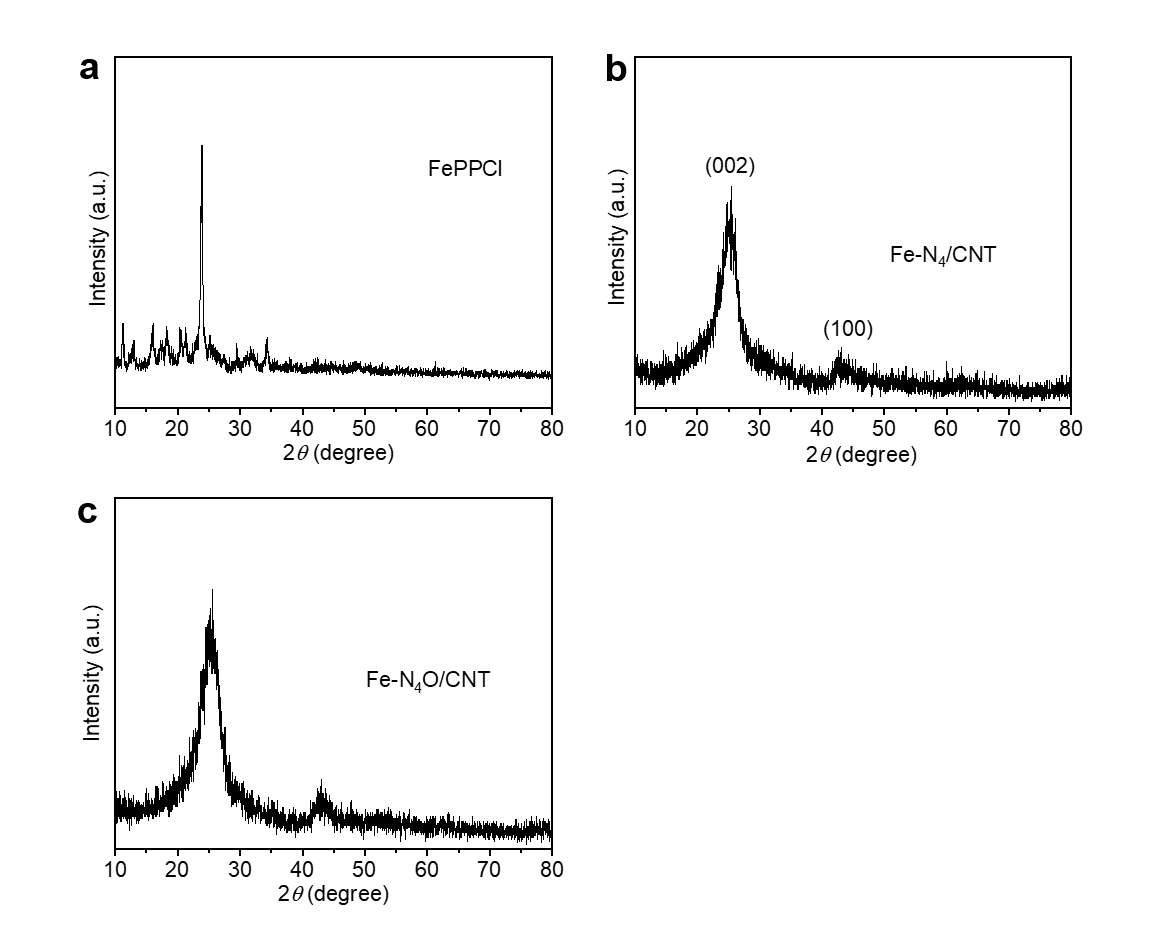


**Figure S2. (a)** XRD pattern of FePPCl. **(b)** XRD pattern of Fe-N_4_/CNT. **(c)** XRD pattern of Fe-N_4_O/CNT. The diffraction features are dominated by the graphitic CNT support, and no discernible reflections from FePPCl and FePc phases are observed in Fe-N_4_O/CNT and Fe-N_4_/CNT, consistent with highly dispersed Fe species.


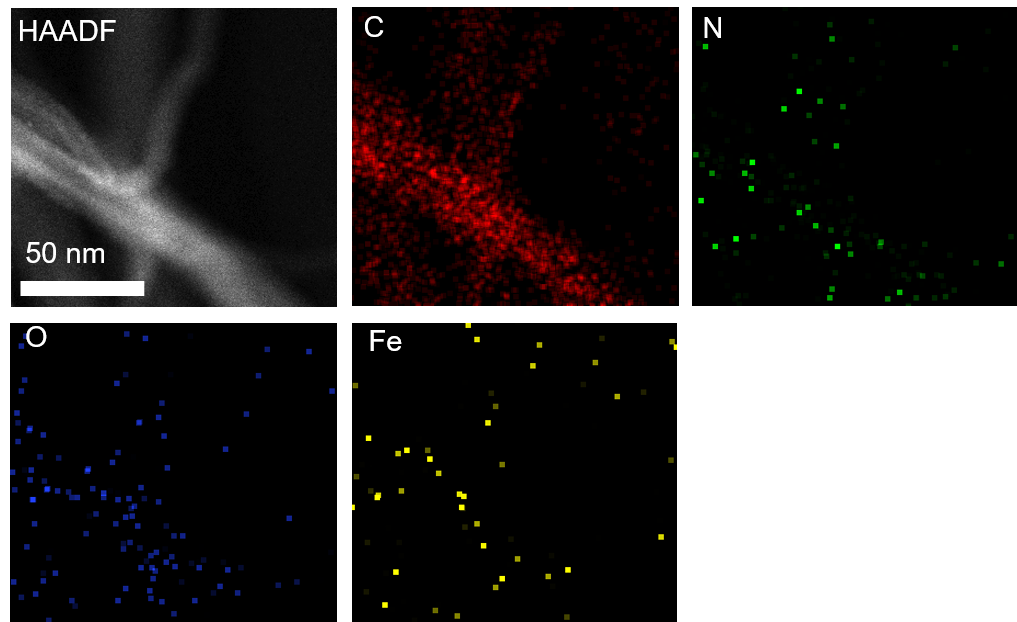


**Figure S3.** HAADF-STEM image and corresponding EDS elemental maps of Fe–N_4_O/CNT. The C map shows the CNT structure, while N, O, and Fe signals are spread across the support without forming clear Fe-rich clusters.


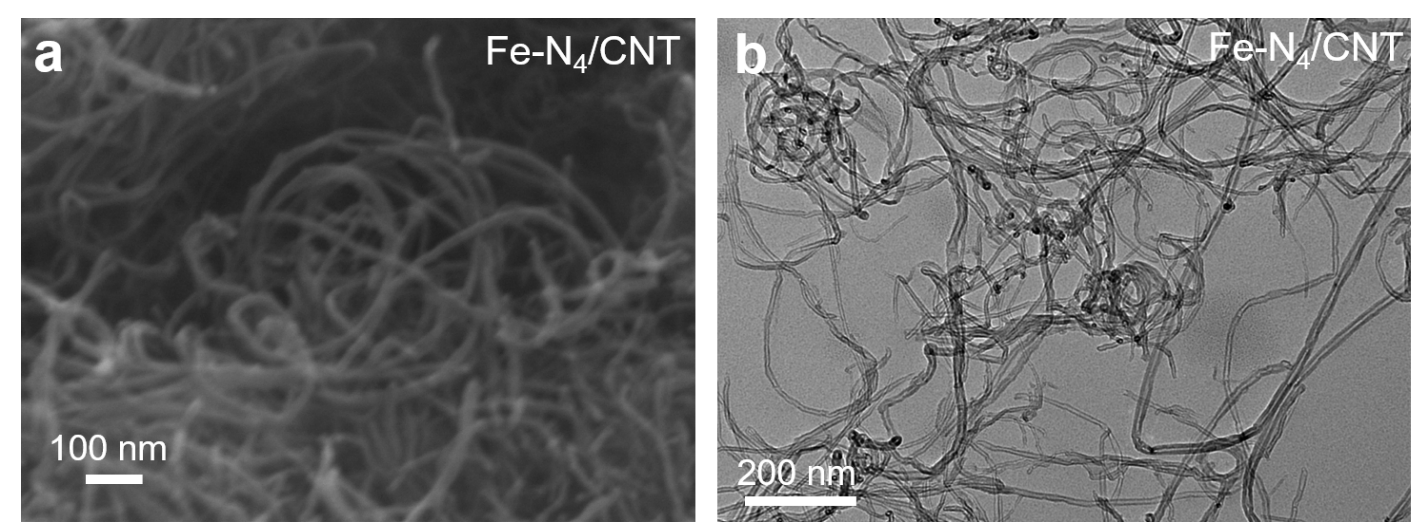


**Figure S4.** (a) SEM image and (b) TEM image of Fe–N_4_/CNT. Both images show an entangled CNT network with preserved tubular morphology after catalyst preparation, and no obvious Fe-containing nanoparticles or agglomerates are observed at these scales.


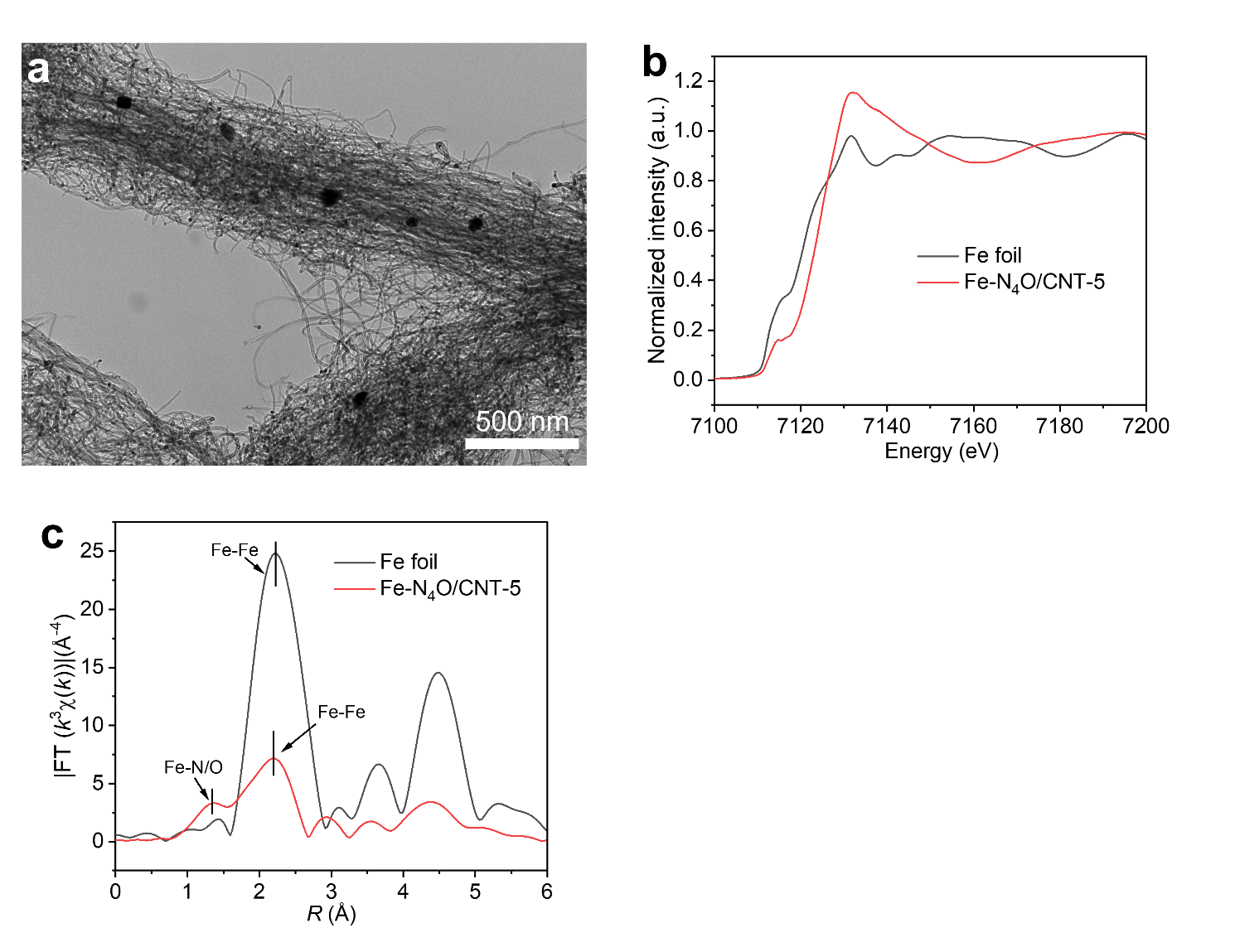


**Figure S5.** TEM and XAS analysis of Fe–N_4_O/CNT-5, showing Fe nanoparticle formation at higher FePPCl loadings. **(a)** TEM image of Fe–N_4_O/CNT-5. **(b)** Experimental Fe K-edge XANES spectra of Fe–N_4_O/CNT-5 and Fe foil. **(c)** Fourier-transformed magnitudes of the Fe K-edge EXAFS signals of Fe–N_4_O/CNT-5 and Fe foil. The TEM and XAS data reveal that increasing the FePPCl amount results in Fe nanoparticle formation after pyrolysis, which decreases the utilization efficiency of Fe species in the catalyst.


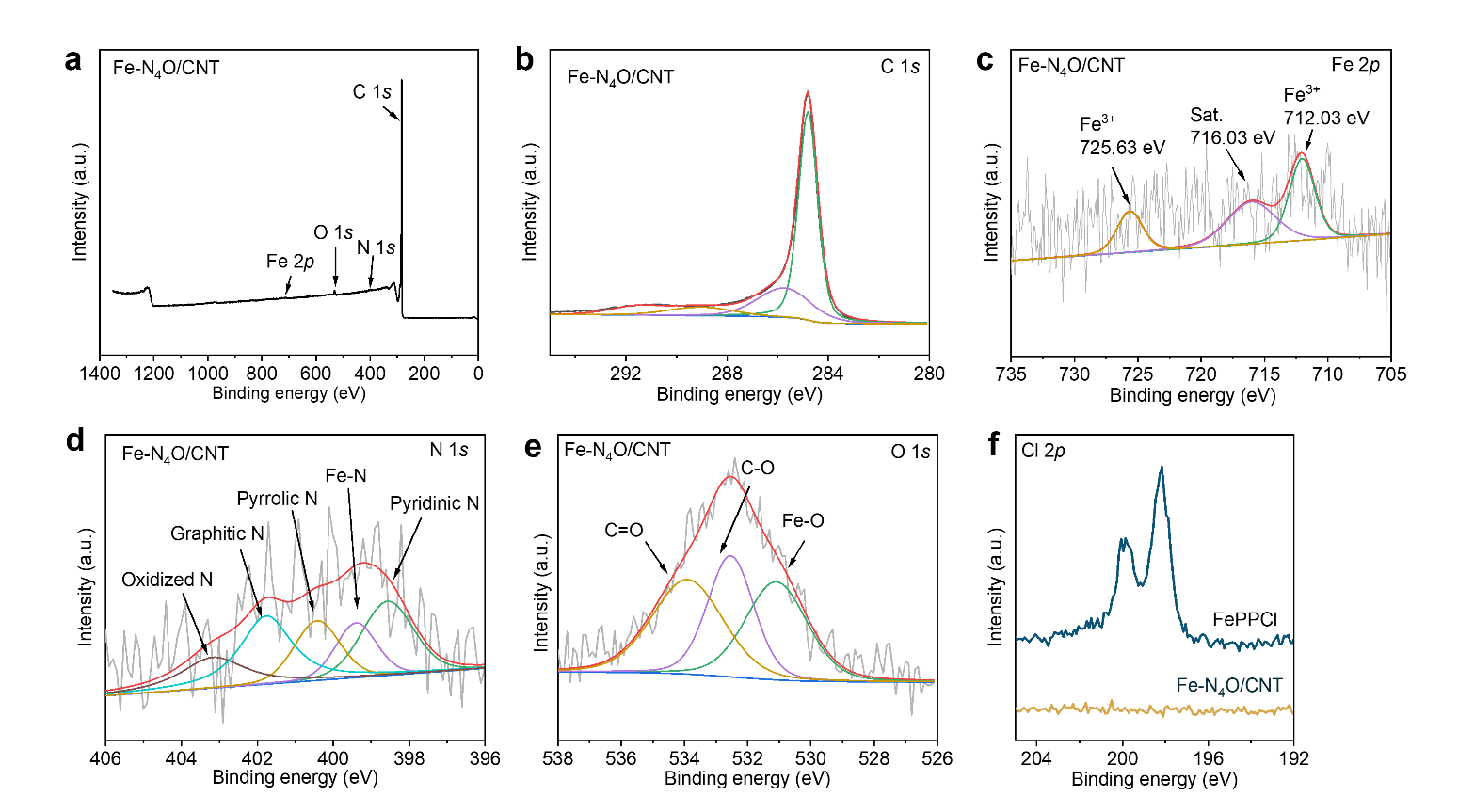


**Figure S6.** (a–f) High-resolution XPS spectra of Fe–N_4_O/CNT. (a) XPS survey spectrum of Fe-N_4_O/CNT. (b) C 1*s* spectrum with peak deconvolution into the major carbon environments of the CNT support. (c) Fe 2*p* spectrum confirming the presence of Fe species in the catalyst. (d) N 1s spectrum deconvoluted into pyridinic N, pyrrolic N, graphitic N, oxidized N, and Fe–Nx (metal–nitrogen) coordination features. (e) O 1*s* spectrum deconvoluted into C=O, C–O, and Fe–O species, indicating oxygen-containing functionalities and Fe–O-related environments in Fe–N_4_O/CNT. (f) Comparison of Cl 2p spectra between Fe–N_4_O/CNT and the FePPCl precursor, showing that the characteristic Cl signal from FePPCl is absent after catalyst synthesis, consistent with removal of the chloride ligand during immobilization/pyrolysis.


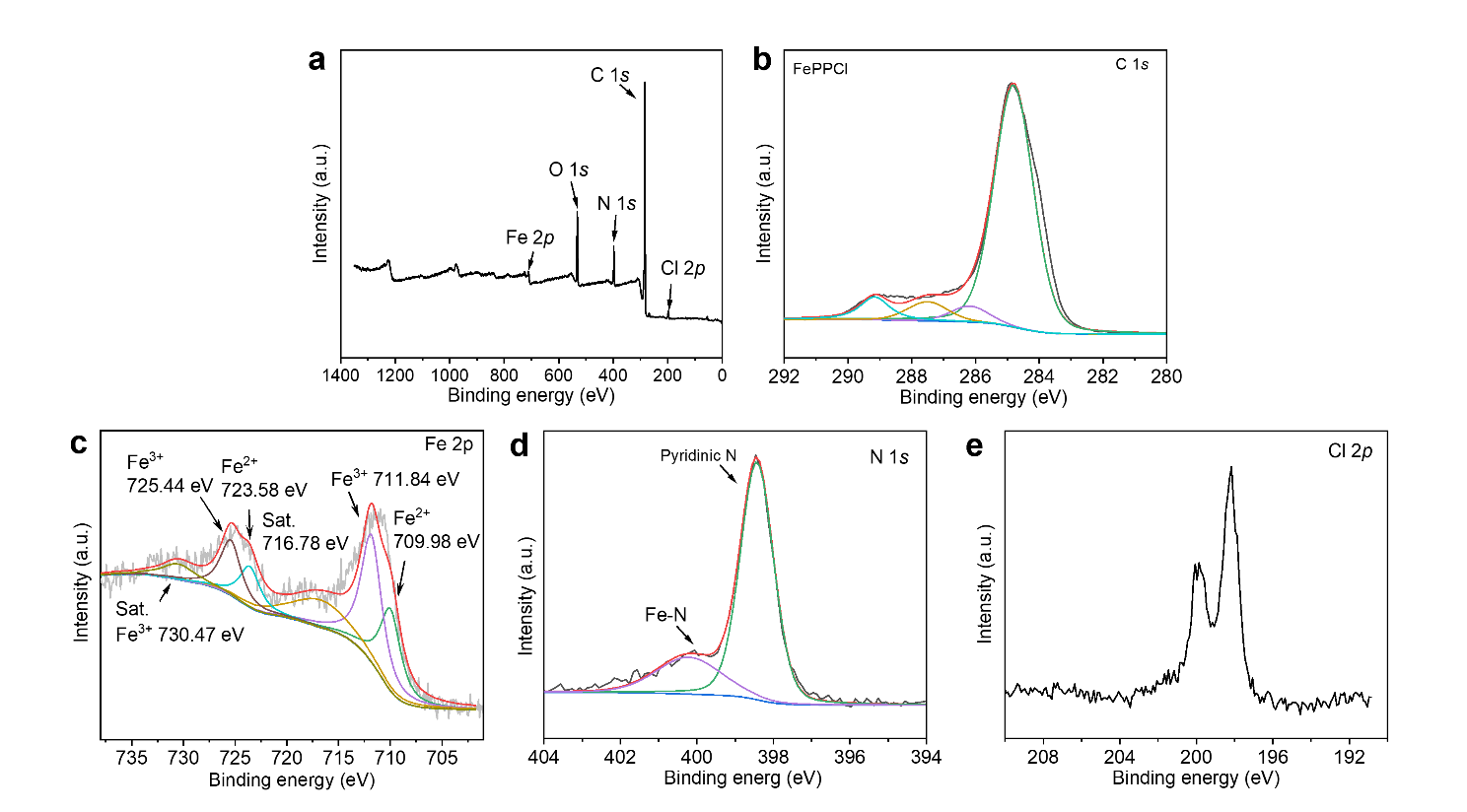


**Figure S7.** (a–e) High-resolution XPS spectra of the FePPCl precursor. (a) XPS survey spectrum of FePPCl. (b) C 1s spectrum showing the carbon environments of the porphyrinic framework. (c) Fe 2*p* spectrum confirming the presence of the Fe center in FePPCl. (d) N 1*s* spectrum characteristic of porphyrinic nitrogen species. (e) Cl 2*p* spectrum exhibiting the characteristic chloride signal typical of the FePPCl precursor.


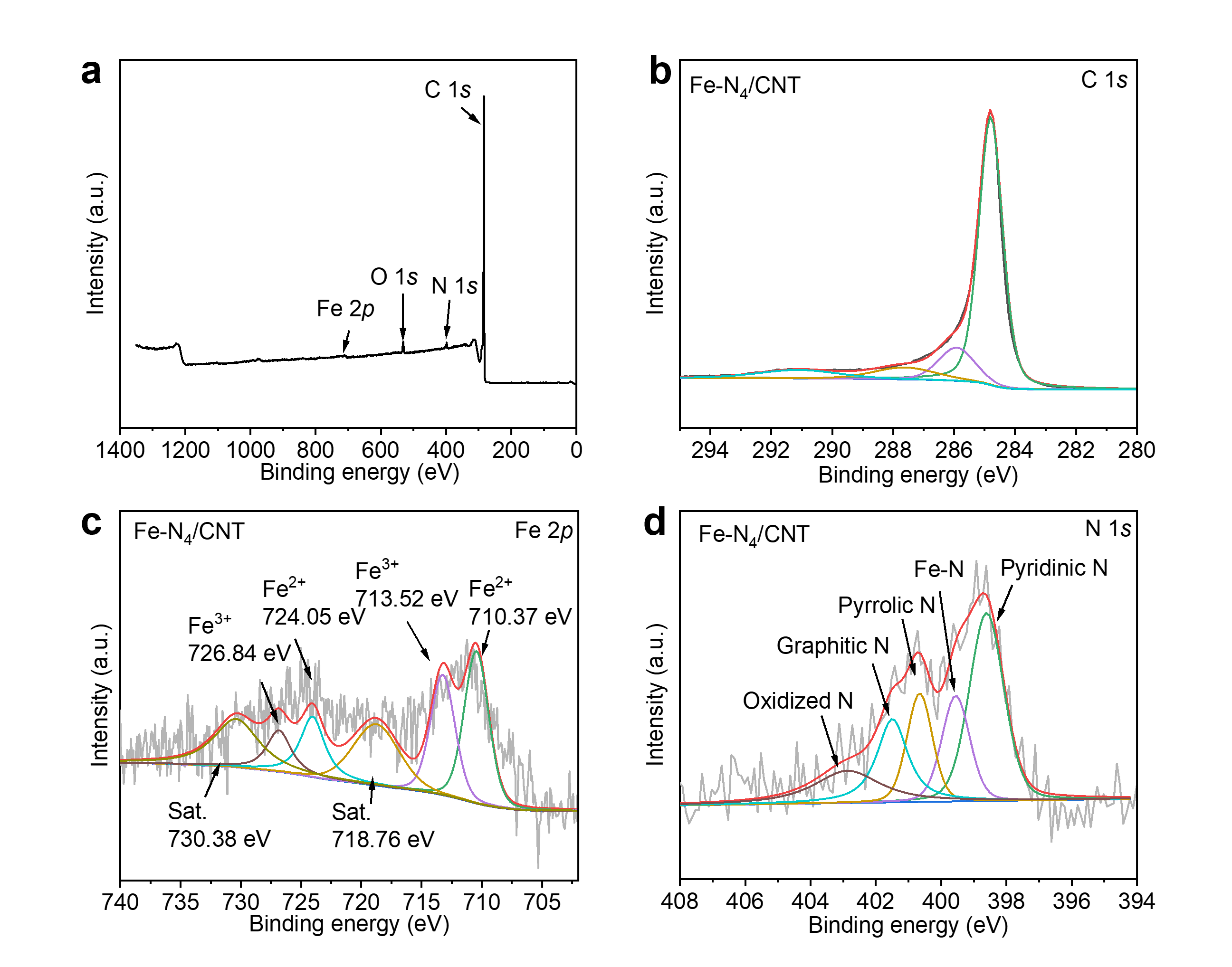


**Figure S8.** (a–d) High-resolution XPS spectra of Fe–N_4_/CNT. (a) XPS survey spectrum of Fe–N_4_/CNT. (b) C 1*s* spectrum showing the different carbon environments in the CNT support. (c) Fe 2*p* spectrum confirming the presence of Fe species within Fe–N_4_/CNT. (d) N 1s spectrum deconvoluted into pyridinic N, pyrrolic N, graphitic N, oxidized N, and Fe-N_x_ (metal-nitrogen) coordination features, consistent with the formation of Fe–N_4_-related sites on CNT.

**Figure S9.** Calculated Fe valence state of Fe–N_4_O/CNT, alongside reference compounds, for comparison with the XPS valence-state analysis. The results from XPS show that the Fe valence state in Fe–N_4_O/CNT is close to +3, aligning with the XPS analysis.


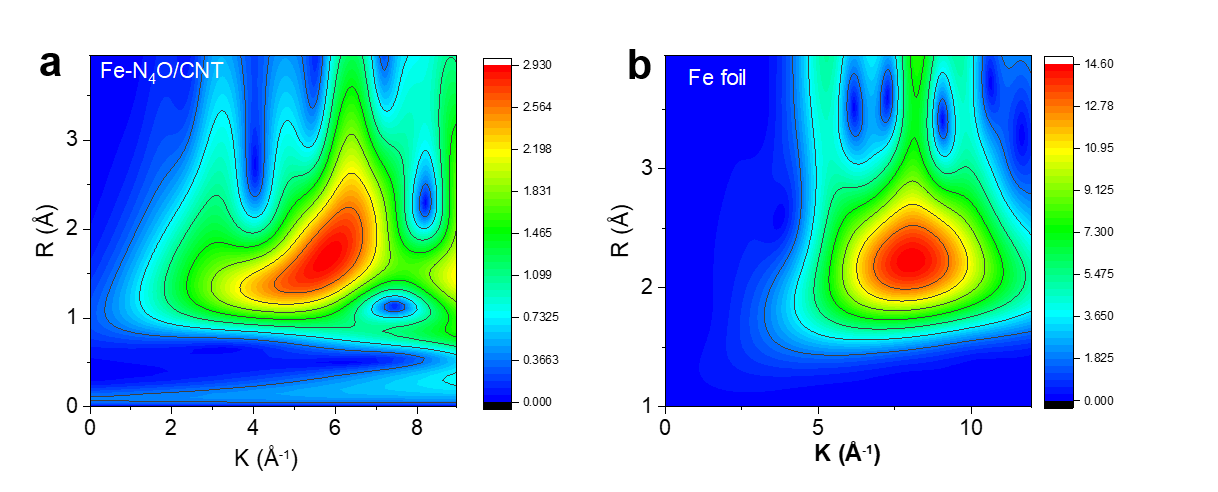


**Figure S10.** (a,b) Wavelet-transform (WT) EXAFS contour plots of Fe–N_4_O/CNT **(a)** and Fe foil **(b)**. The WT-EXAFS map of Fe–N_4_O/CNT displays a dominant feature characteristic of light-atom (N/O) coordination in the first shell, while Fe foil shows an intense feature typical of Fe–Fe scattering. This serves as a reference to differentiate single Fe site coordination from metallic Fe–Fe contributions.


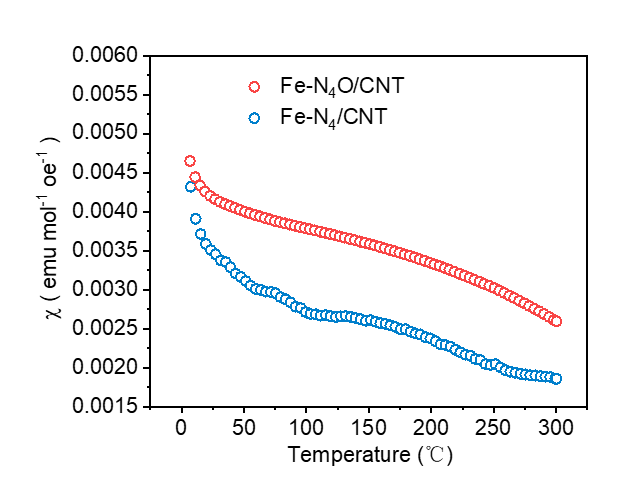


**Figure S11.** Magnetic susceptibility–temperature (𝜒-T) plots of Fe–N_4_/CNT and Fe–N_4_O/CNT measured under the same conditions, illustrating the temperature-dependent magnetic response of both catalysts.


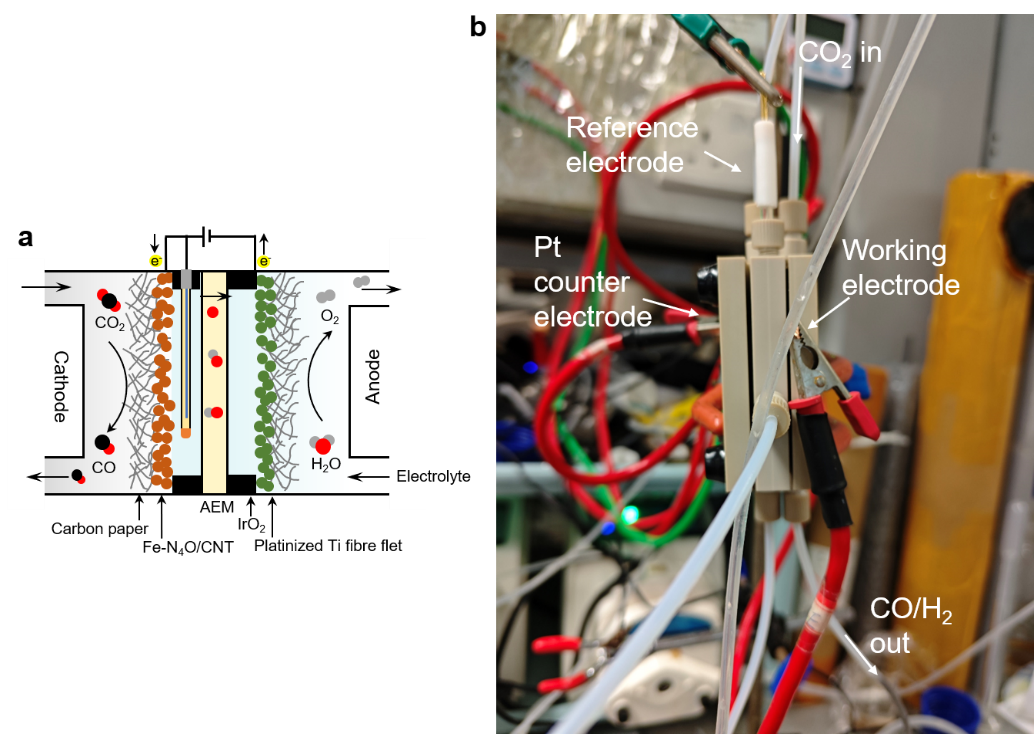


**Figure S12.** **(a)** Schematic diagram of the flow-cell setup and its working principle. CO_2_ is supplied to the gas-diffusion cathode, where CO is produced and leaves with the gas stream, while O_2_ forms at the anode in the liquid electrolyte chamber. The two chambers are separated by an ion-exchange membrane to ensure ionic conduction and product separation. **(b)** Photo of the assembled flow-cell system, showing the working electrode, Pt counter electrode, reference electrode, CO_2_ inlet, and CO/H_2_ outlet.

**Figure S13.** Faradaic efficiency toward CO of Fe–N_4_O/CNT-5 at different applied current densities in 0.5 M K_2_SO_4_ (pH=2), showing decreased CO selectivity under acidic conditions due to Fe nanoparticle formation at higher FePPCl loadings. Faradaic efficiency toward CO of Fe–N_4_O/CNT-5 at various applied current densities, determined from the outlet gas composition during steady-state electrolysis at each operating point. The decline in CO Faradaic efficiency under acidic conditions is attributed to Fe nanoparticle formation during pyrolysis with increased FePPCl loading, which reduces the number of atomically dispersed active Fe sites.


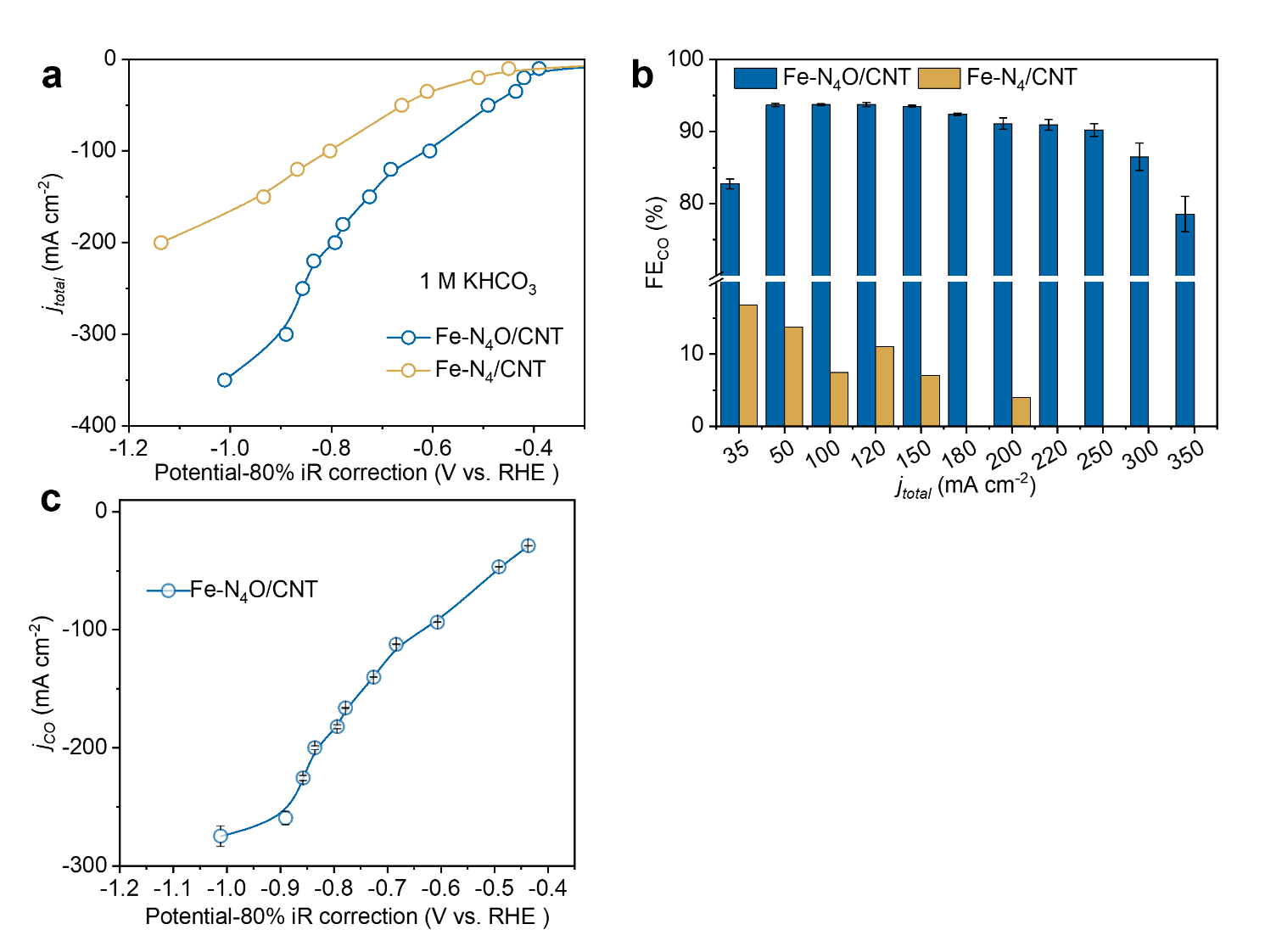


**Figure S14. (a)** Polarization (I–V) curves of Fe–N_4_O/CNT and Fe–N_4_/CNT recorded in CO_2_-saturated 1.0 M KHCO_3_ using the flow-cell setup (current density normalized to the geometric electrode area). **(b)** Faradaic efficiency for CO as a function of different applied current densities, determined from the outlet gas composition during steady-state electrolysis at each operating point. **(c)** Partial current density for CO formation (*j*_CO_), calculated from the total current density **(j)** and FE_CO_. Error bars indicate the standard deviation of replicate measurements. As shown in Figure S14a and b, Fe–N_4_O/CNT demonstrates higher CO_2_RR activity than Fe–N_4_/CNT under neutral conditions, evidenced by its improved polarization behavior and higher FE_CO_ at comparable operating conditions.


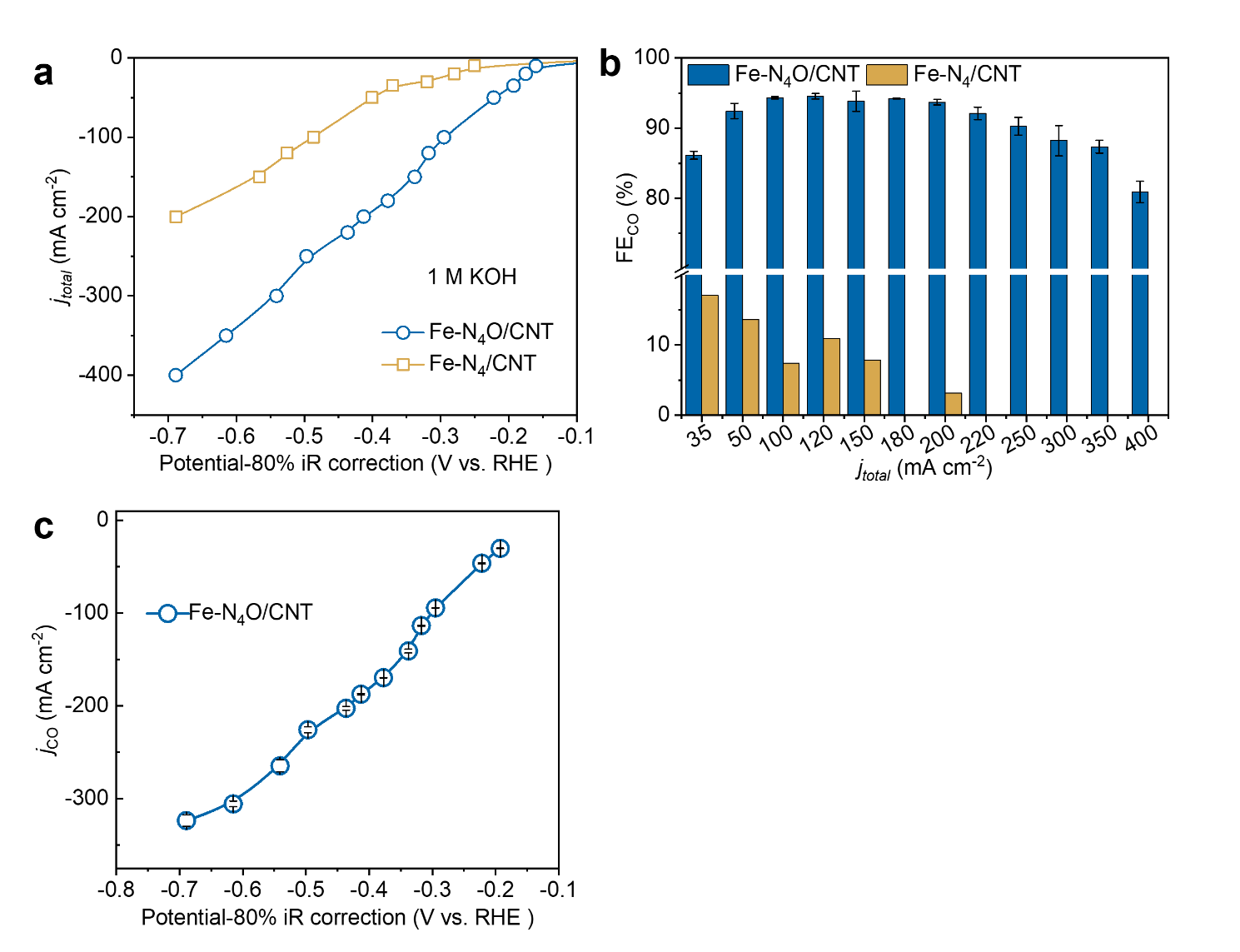


**Figure S15.** **(a)** Polarization (I–V) curves of Fe–N_4_O/CNT and Fe–N_4_/CNT recorded in CO_2_-fed 1.0 M KOH using the flow-cell setup (current density normalized to the geometric electrode area). **(b)** Faradaic efficiency for CO of Fe–N_4_O/CNT and Fe–N_4_/CNT at different applied current densities, determined from the outlet gas composition during steady-state electrolysis at each operating point. **(c)** Partial current density for CO formation (*j*_CO_) calculated from the total current density (j) and FE_CO_. Error bars indicate the standard deviation of replicate measurements. As shown in Figure S15a and b, Fe–N_4_O/CNT displays higher CO_2_RR activity than Fe–N_4_/CNT in alkaline conditions, as evidenced by its improved polarization behavior and higher FE_CO_ under similar operating conditions.

**Figure S16.** Turnover frequency for CO formation (TOF_CO_) of Fe–N_4_O/CNT and Fe–N_4_/CNT measured in 0.5 M K_2_SO_4_ with added H_2_SO_4_. TOF_CO_ values were calculated from the CO partial current (*j*_CO_) and normalized to the amount of catalytically accessible Fe sites, determined from Fe loading and active-site quantification (see Methods). This provides an intrinsic-activity comparison under the same acidic electrolyte conditions.


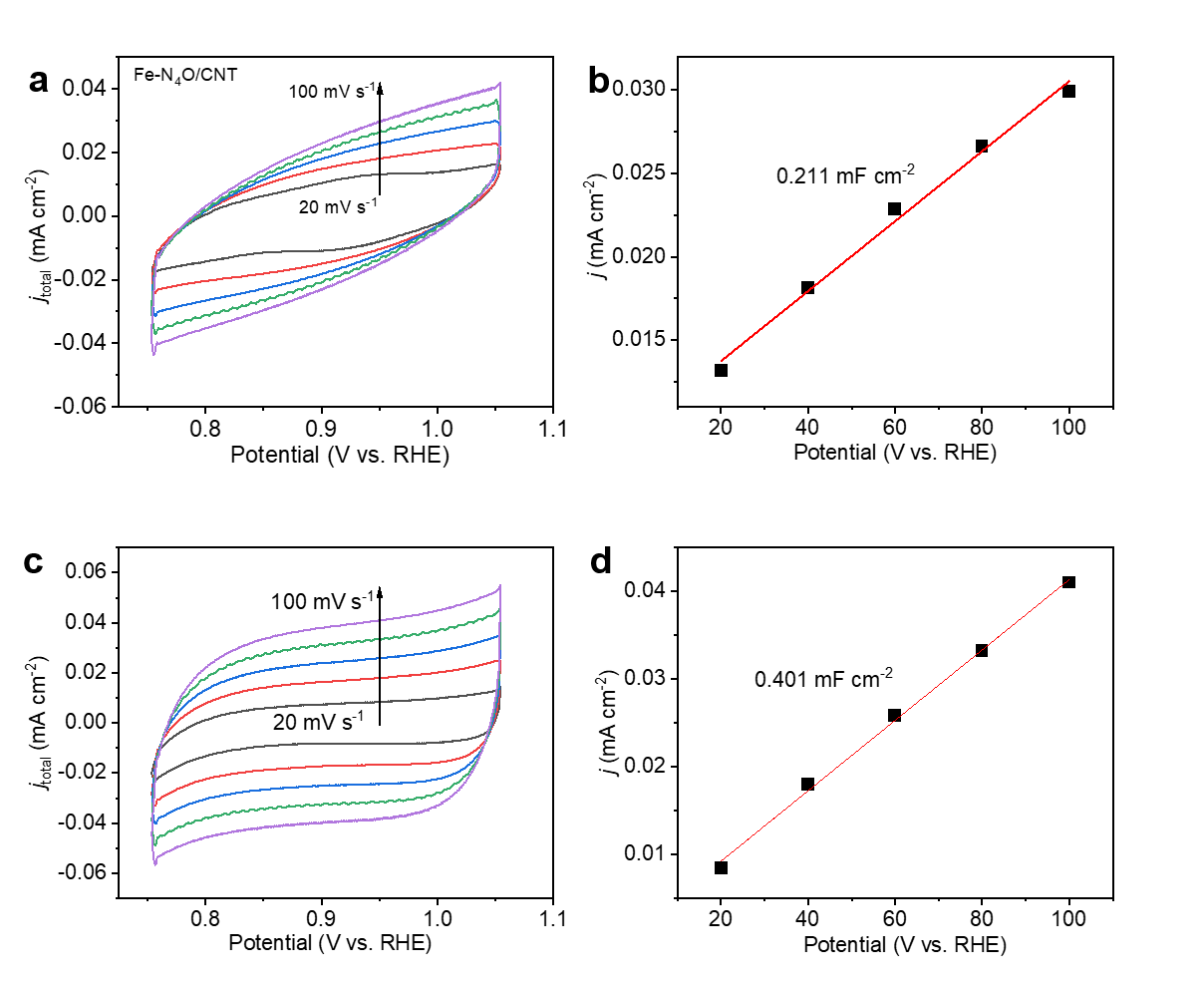


**Figure S17.** **(a,c)** Cyclic voltammograms (CVs) of Fe–N_4_O/CNT (a) and Fe–N_4_/CNT (c) recorded within a non-faradaic potential window at various scan rates. (b,d) Calculation of the double-layer capacitance (C_dl_) for Fe–N_4_O/CNT **(b)** and Fe–N_4_/CNT **(d)** by linearly fitting the capacitive current (j) as a function of scan rate. The obtained C_dl_ values are used to estimate the relative electrochemically active surface area (ECSA)[15].

**Figure S18.** ECSA-normalized partial current density for CO production (j(CO)_ECSA_) of Fe–N_4_O/CNT and Fe–N_4_/CNT. The *j*_CO_ values were normalized by the electrochemically active surface area (ECSA) estimated from the double-layer capacitance (C_dl_) measurements (Figure S17), allowing comparison of the intrinsic CO formation activity regardless of differences in surface area.

**Figure S19.** Faradaic efficiency toward CO of Fe–N_4_O/CNT at different applied current densities in 1 M KHCO_3_ and 1 M KHCO_3_ + 0.05 M KSCN. The addition of SCN⁻ significantly reduced the CO selectivity of Fe–N_4_O/CNT across the entire current-density range. Notably, the FE_CO_ decreased to 38.42% at 350 mA cm^-2^ in 1 M KHCO_3_ + 0.05 M KSCN, indicating effective poisoning of the Fe center and supporting that Fe–N_4_O is the true active site for CO_2_RR.


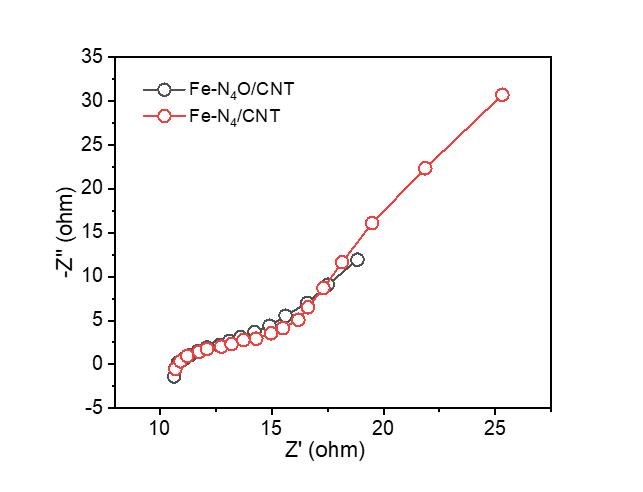


**Figure S20.** Electrochemical impedance spectroscopy (EIS) Nyquist plots of Fe–N_4_O/CNT and Fe–N_4_/CNT measured under identical conditions. The high-to-medium frequency semicircle indicates the charge-transfer resistance (Rct) at the electrode–electrolyte interface, allowing comparison of interfacial charge-transfer kinetics between the two catalysts.


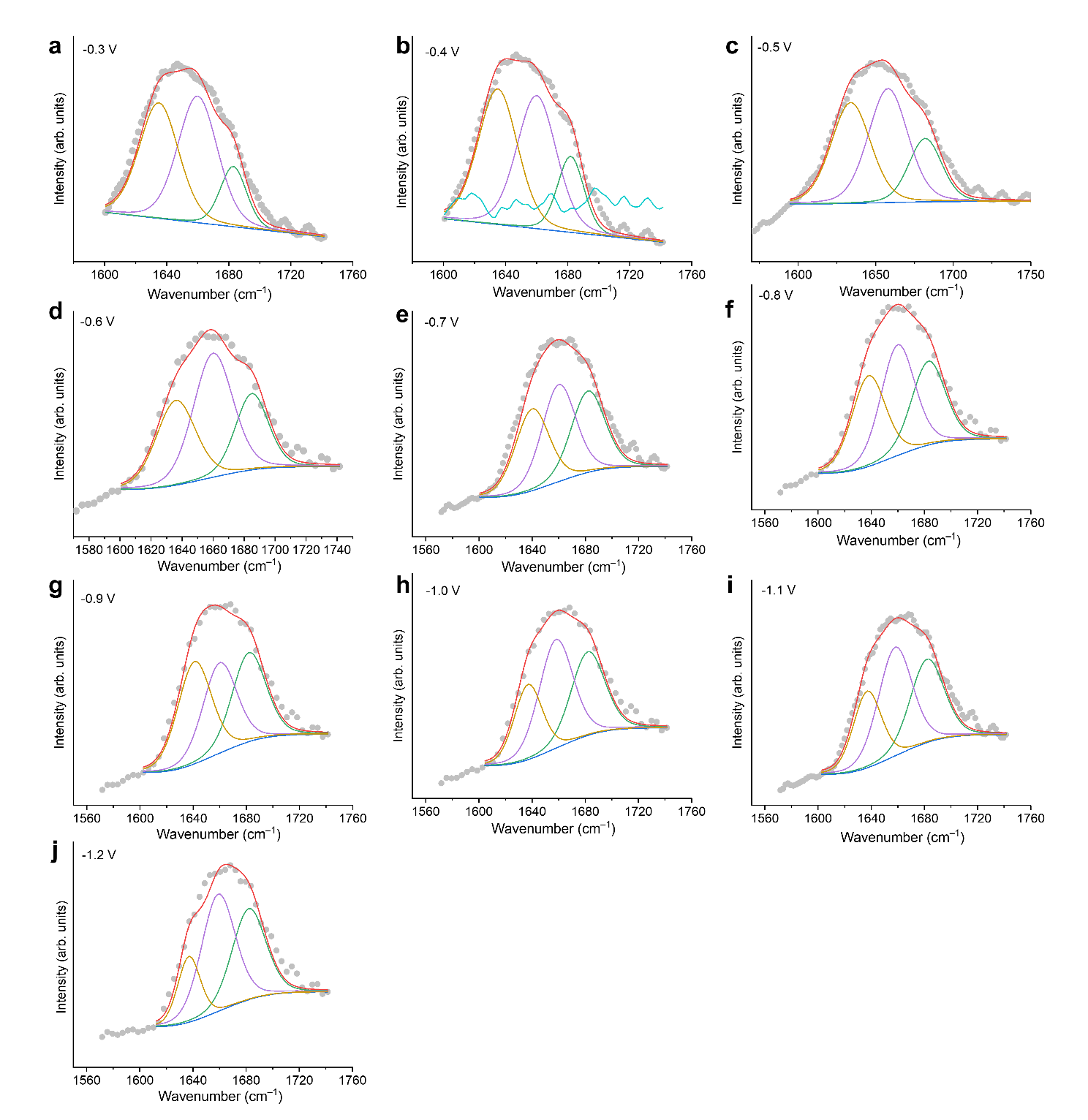


**Figure S21.** (a–j) In situ ATR-SEIRAS spectra of the interfacial water region collected on Fe–N_4_/CNT at various applied potentials in CO_2_-saturated 0.5 M K_2_SO_4_ with H_2_SO_4_ added (pH = 2). The O-H stretching region was deconvoluted into multiple components to monitor the potential-dependent changes of interfacial water species at the catalyst surface.


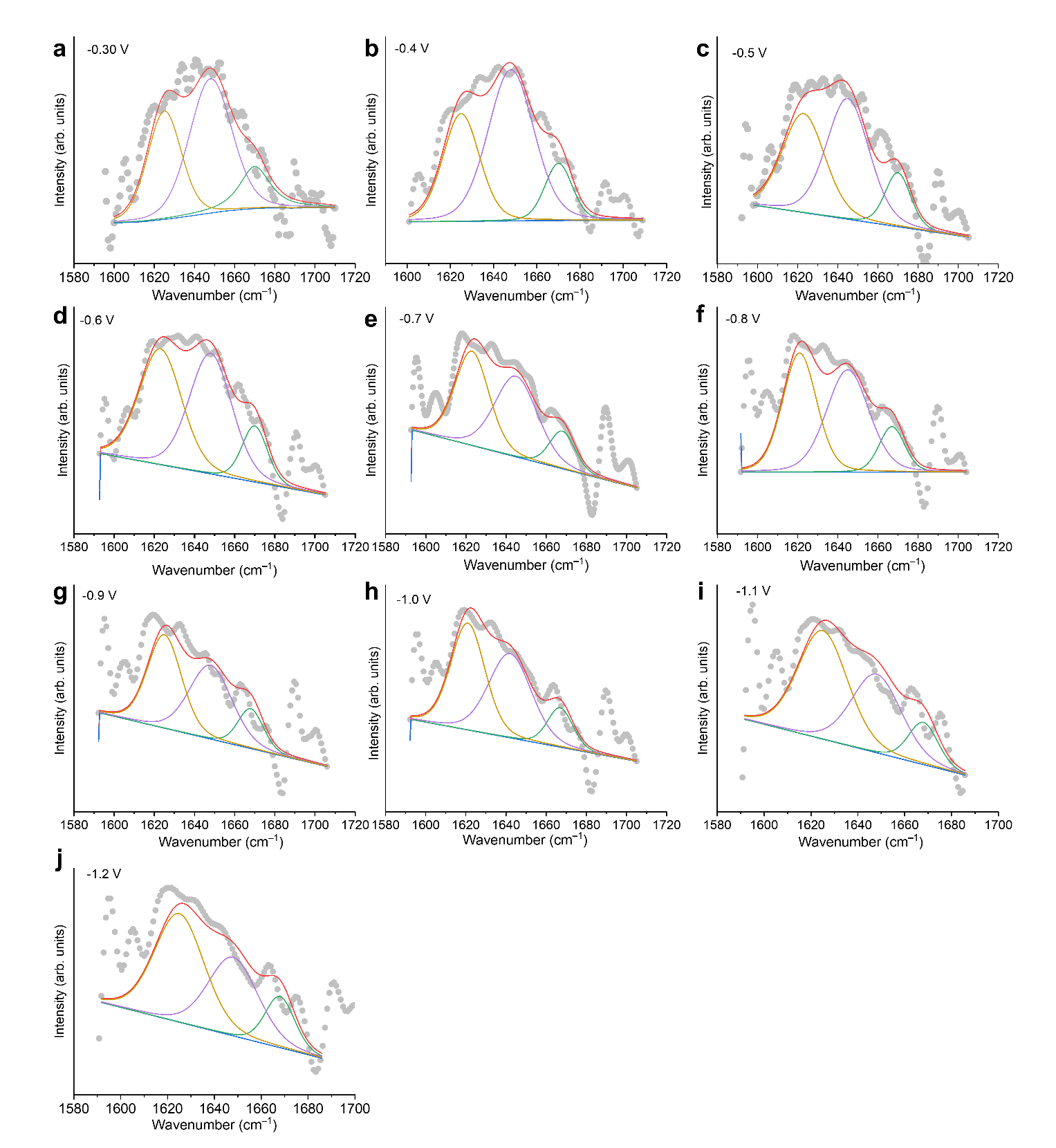


**Figure S22.** (a–j) In situ ATR-SEIRAS spectra of the interfacial water region collected on Fe–N_4_O/CNT at different applied potentials in CO_2_-saturated 0.5 M K_2_SO_4_ with added H_2_SO_4_ (pH = 2). The O-H stretching region was deconvoluted into multiple components to track the potential-dependent changes of interfacial water species at the catalyst surface.

**Figure S23.** LSV curves of Fe-N_4_/CNT **(a)** and Fe-N_4_O/CNT **(b)** recorded in Ar-saturated 0.5 M K_2_SO_4_ electrolyte (pH = 2) at different rotation rates using a rotating disk electrode. **(c)** Comparison of the LSV curves of Fe-N_4_/CNT and Fe-N_4_O/CNT at 1,000 rpm. All data were collected without iR compensation. The HER behaviors of Fe-N_4_/CNT and Fe-N_4_O/CNT were studied in acidic 0.5 M K_2_SO_4_ electrolyte (pH = 2) with a rotating disk electrode (Figure S23a-b). The HER onset potentials of both catalysts are similar. For both, a current density plateau appears, indicating diffusion-limited hydronium reduction. Compared to Fe-N_4_/CNT, Fe-N_4_O/CNT shows a lower plateau current density (Figure S23c), possibly because the intermediate-spin Fe sites reduce the attraction to H_3_O^+^, thus suppressing hydronium buildup and the HER process.

**Figure S24.** Linear fitting of *j*_plateau_ vs. *ω*^1/2^ for (a) Fe-N_4_/CNT and (b) Fe-N_4_O/CNT according to the Levich equation. Fe-N_4_O/CNT shows a smaller slope (0.53) than Fe-N_4_/CNT (0.65), indicating that the intermediate-spin Fe sites reduce the diffusion-limited electroreduction of H_3_O^+^. Additionally, the H_3_O^+^ diffusion coefficient (D_H3O_^+^) was calculated for each sample. Compared with Fe-N_4_/CNT, Fe-N_4_O/CNT has approximately 26.44% lower D_H3O_^+^, suggesting that the intermediate-spin Fe sites are less favorable for H_3_O^+^ adsorption, thus suppressing the HER process.


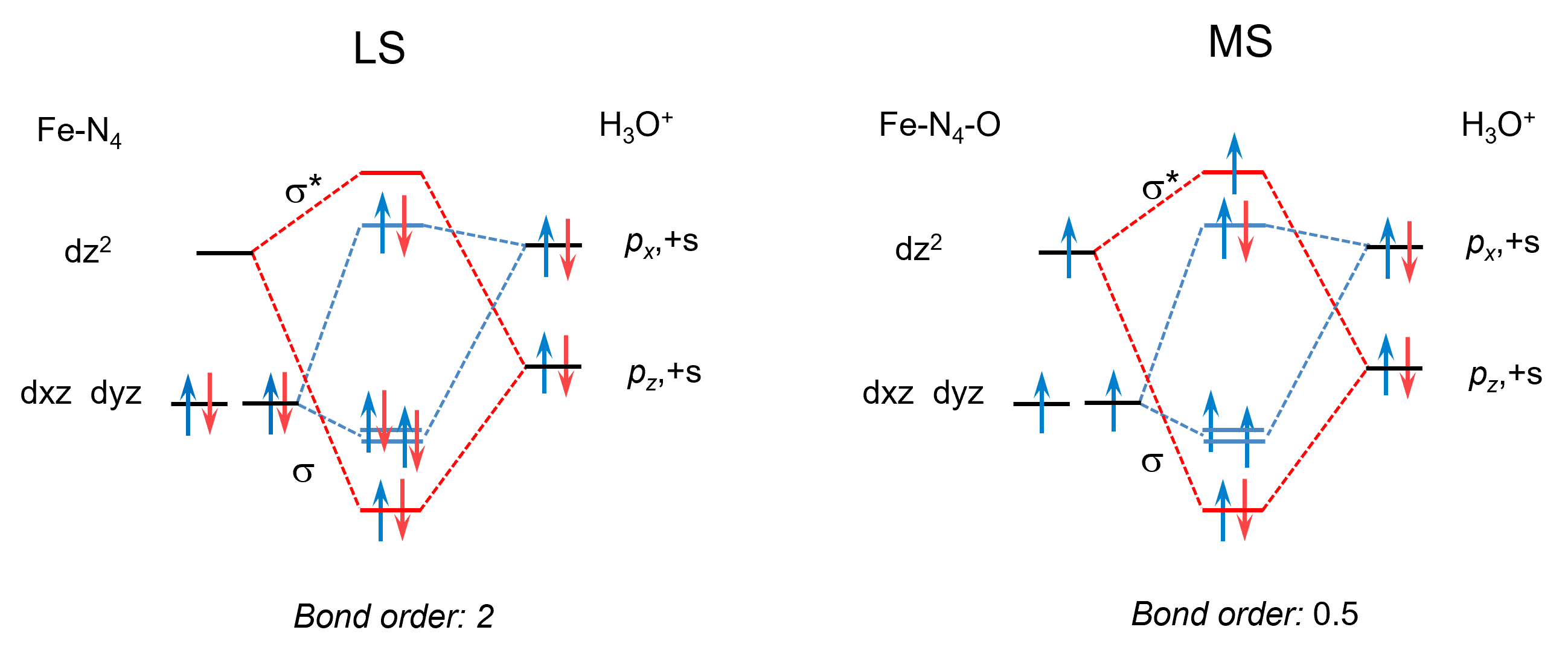


**Figure S25.** (a,b) Schematic orbital interaction diagrams illustrating the electronic coupling between H_3_O^+^ and the Fe center in Fe-N_4_ (a) and Fe-N_4_O (b). The diagrams compare the relative alignment and interaction of the relevant Fe-centered orbitals with the frontier orbitals of H_3_O^+^, highlighting how spin optimization in Fe-N_4_O modulates the Fe-H_3_O^+^ interaction at the interface.


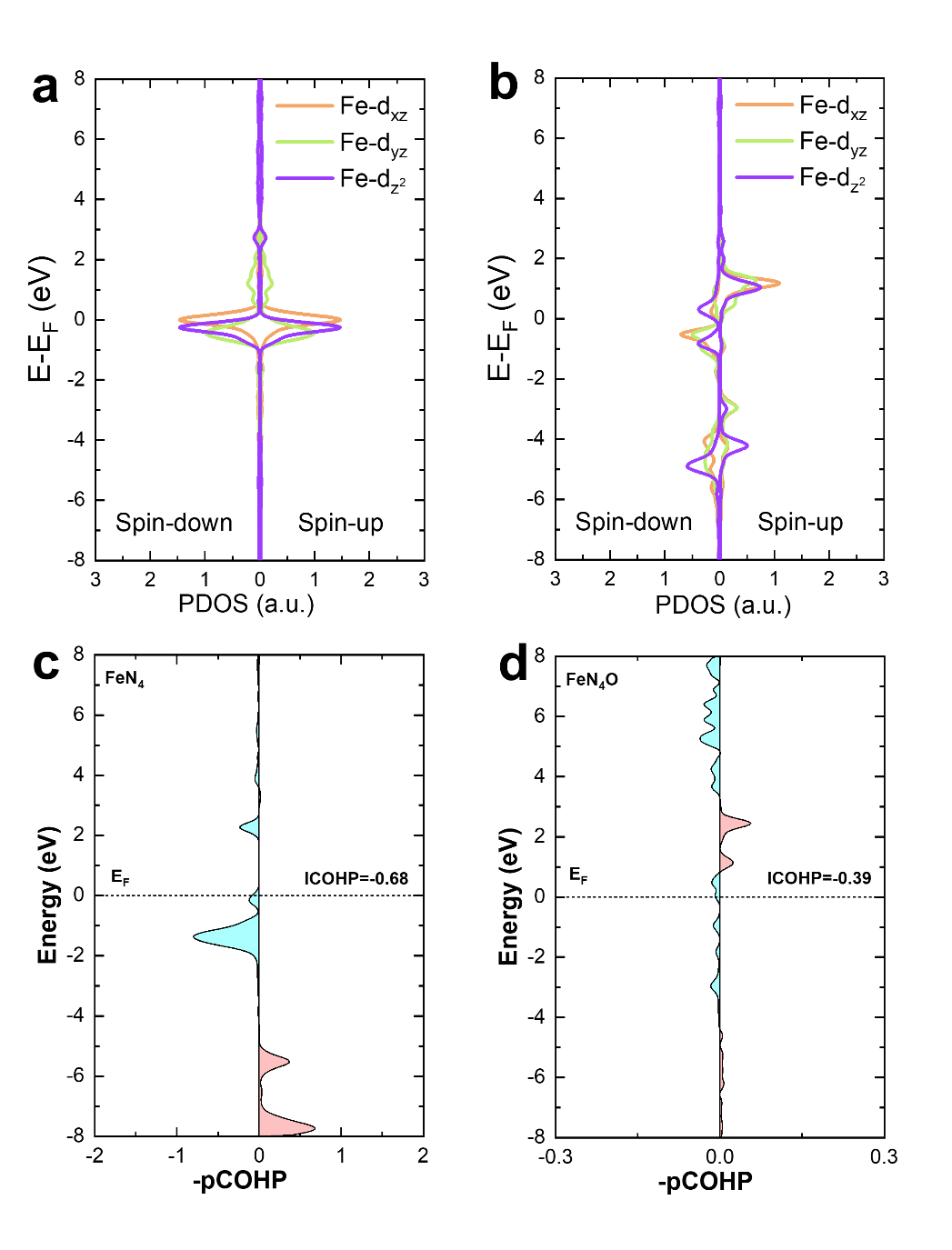


**Figure S26.** Projected density of states (PDOS) of the Fe-3*d* orbital for FeN_4_-H_2_O (a) and FeN_4_O-H_2_O (b). E-E_f_ represents energy relative to the Fermi level, which is set at zero. Projected crystal orbital Hamilton population (pCOHP) of Fe-O bonds in FeN_4_-H_2_O (c) and FeN_4_O-H_2_O (d). Bonding and antibonding contributions are indicated by positive and negative -pCOHP values, respectively. The density of states (DOS) results (Figure S26a-b) show that, compared with Fe-N_4_O, the *d*-band center of Fe-N_4_ is closer to the Fermi level, suggesting stronger H_2_O adsorption on Fe-N_4_. In comparison to Fe-N_4_-H_2_O (ICOHP: −0.68, Figures S26c), the pCOHP of Fe-N_4_O-H_2_O decreases and shifts positively (ICOHP: −0.39, Figures S26d), indicating that the Fe-O bonds in Fe-N_4_O-H_2_O are significantly weaker than those in Fe-N_4_-H_2_O.


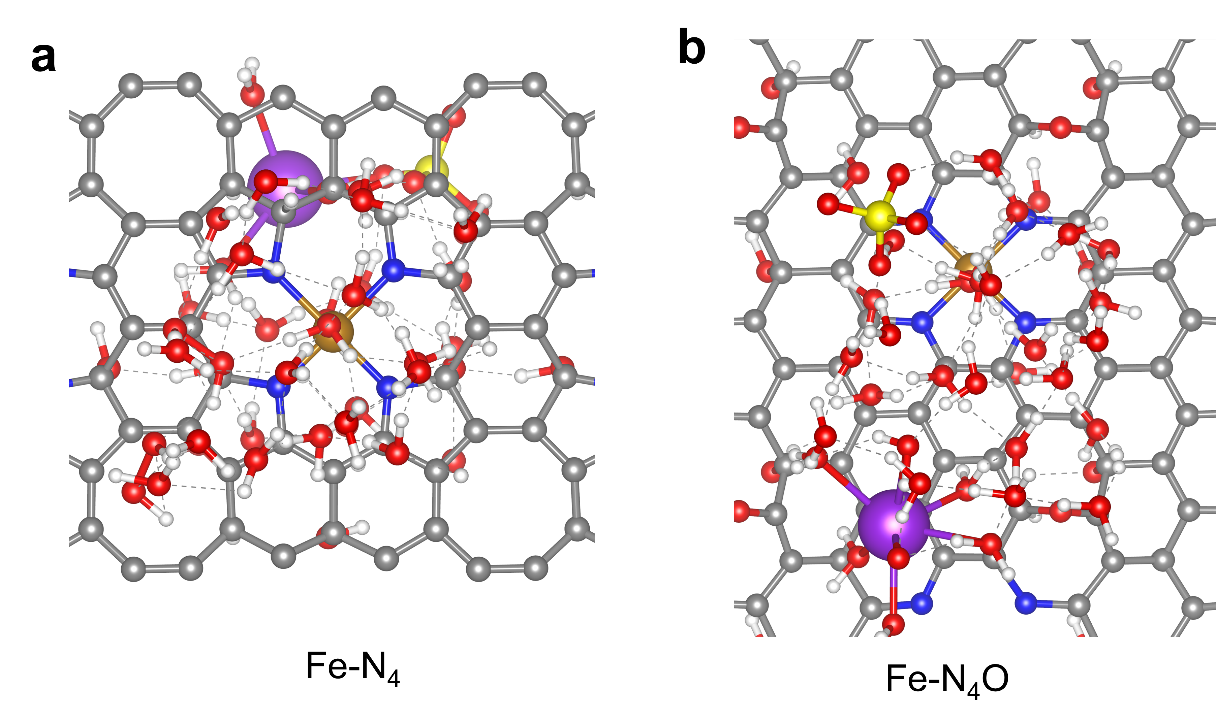


**Figure S27.** A typical model of Fe-N_4_O **(a)** and Fe-N_4_ **(b)** for MD simulations, red: O, white: H, blue: N, orange: Fe, purple: K^+^ and yellow: S, and grey: C.

**Figure S28.** Integrated peak area of the CO adsorption band (CO) on Fe-N_4_O/CNT and Fe-N_4_/CNT extracted from in situ IR (ATR-SEIRAS) spectra at different applied potentials. The band area was obtained by baseline correction and peak integration, providing a relative measure of *CO surface coverage under identical conditions.

**Figure S29.** CO temperature-programmed desorption (CO-TPD) profiles of Fe-N_4_O/CNT and Fe-N_4_/CNT. The desorption peaks at different temperatures indicate CO binding with varying strengths on the two catalysts, with higher desorption temperatures signifying stronger CO adsorption.


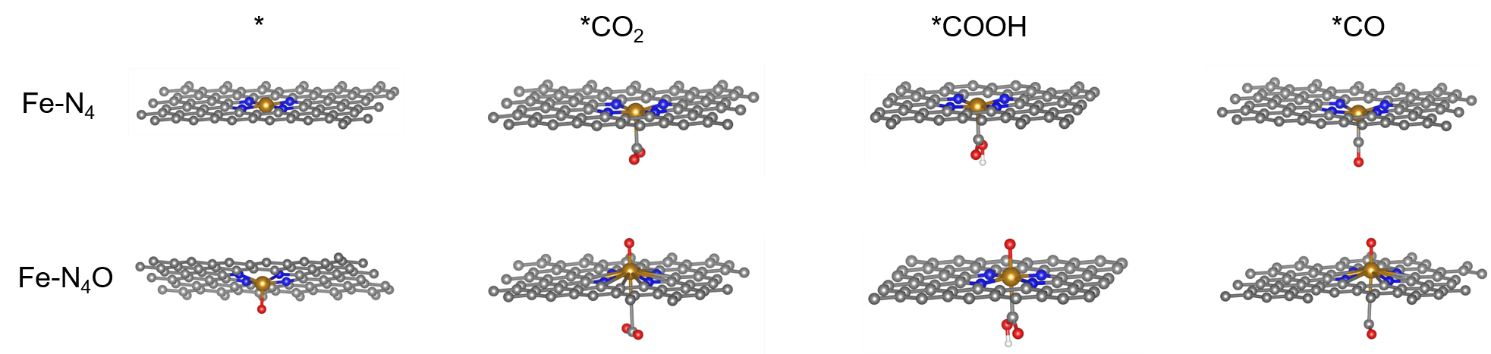


**Figure S30.** DFT-optimized structures of representative CO_2_RR intermediates adsorbed on Fe-N_4_ and Fe-N_4_O active sites. The geometries were obtained after full structural relaxation, illustrating the adsorption configurations and bonding motifs of key intermediates on the two catalysts. Color code: Fe (brown), C (grey), O (red), N (blue), and H (white).


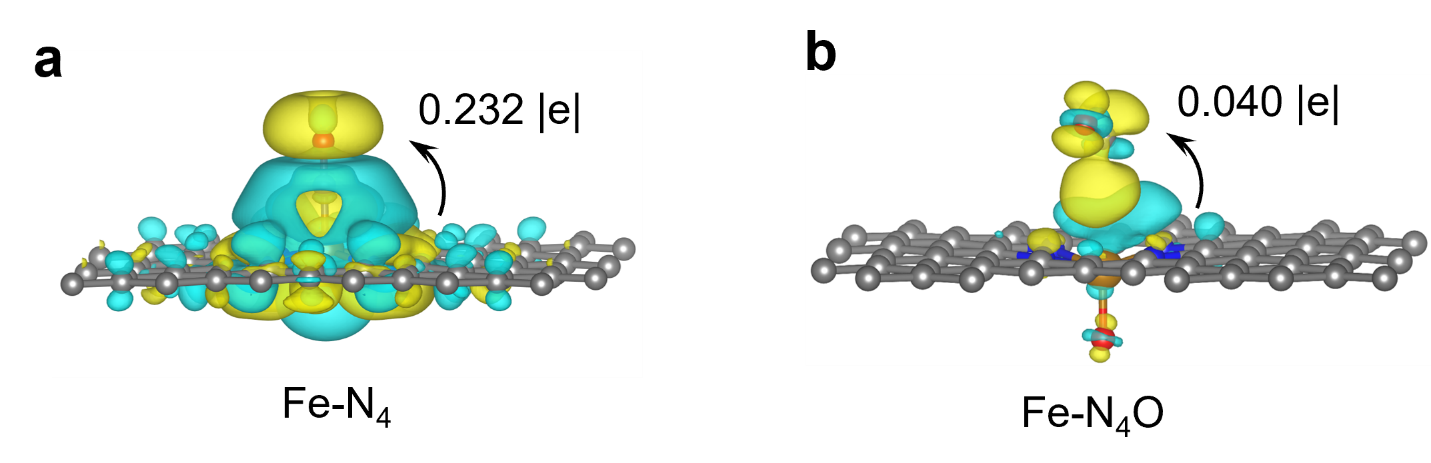


**Figure S31.** Charge density difference distribution between CO and (a) Fe-N_4_, (b) Fe-N_4_O. We compared the electron contribution of Fe-N_4_ (Figure S31a) and Fe-N_4_O (Figure S31b) to CO using charge density difference analysis. The Fe-N_4_ model showed a higher electron contribution to CO (0.232 |e|) than the Fe-N_4_O model (0.04 |e|), indicating a much weaker interaction between Fe-N_4_O and CO compared to Fe-N_4_.





**Figure S32.** Projected density of states (PDOS) of the Fe-3*d* orbital for Fe-N_4_ and Fe-N_4_O. The Fermi level is set to zero. The *d*-band center positions of Fe-N_4_ and Fe-N_4_O are indicated by blue and pink dotted lines, respectively. Density of states (DOS) results (Figure S32) show that the *d*-band center of Fe-N_4_ (-0.338 eV) is close to the Fermi level. In contrast, the *d*-band center of Fe-N_4_O (-1.366 eV) is further from the Fermi level, resulting in weaker CO adsorption and facilitating CO desorption.


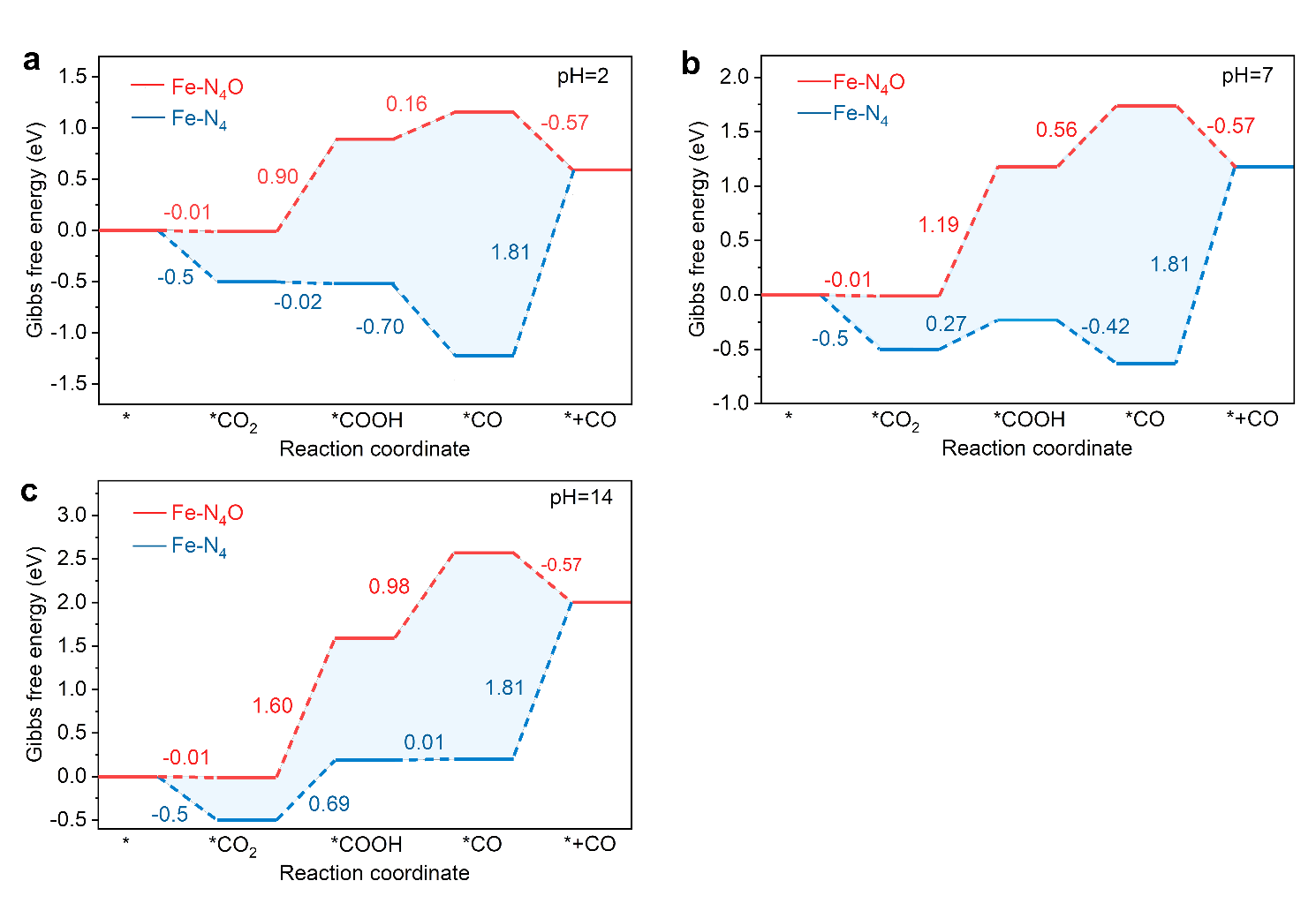


**Figure S33.** Gibbs free energy diagrams of CO_2_ to CO over Fe-N_4_O and Fe-N_4_ models with the free energy correction at pH= 2, 7, and 14, respectively. To better understand the role of axial oxygen in affecting the spin state of Fe single-atom catalysts, additional DFT calculations were performed using the Fe-N_4_ and Fe-N_4_O models under different electrochemical conditions (Figure S33). The CO_2_-to-CO conversion proceeds via a two-electron transfer mechanism involving the adsorption of *CO_2_, *COOH, and *CO intermediates. As shown in Figure S33a, the formation of *COOH (*CO_2_ → *COOH) is endothermic on Fe–N_4_O, requiring over 0.90 eV after pH correction, and is identified as RDS. In contrast, Fe–N_4_ requires more energy (1.81 eV) to overcome the *CO desorption barrier. Under acidic conditions (pH = 2), CO_2_ adsorption on Fe sites in both models is thermodynamically favorable. For Fe–N_4_O, the energy barriers for *CO_2_-to-*COOH and *COOH-to-*CO are 0.90 eV and 0.16 eV, respectively, while *CO desorption is spontaneous (–0.57 eV). Conversely, in Fe–N_4_, the barrier energy for *CO_2_-to-*COOH and *COOH-to-*CO are energetically favorable, but *CO desorption requires a much higher energy barrier. The RDS of Fe–N_4_O shifts to the *CO_2_ hydrogenation step, with corresponding energy barriers of 0.90, 1.19, and 1.60 eV at pH = 2, 7, and 14, respectively, all lower than the 1.81 eV barrier seen in Fe–N_4_. These results show that spin-state modulation changes the occupancy of electronic orbitals, thus affecting the orbital hybridization between intermediates and active sites. This, in turn, influences the interaction strength between the sites and intermediates, ultimately impacting the CO_2_RR performance of the site.


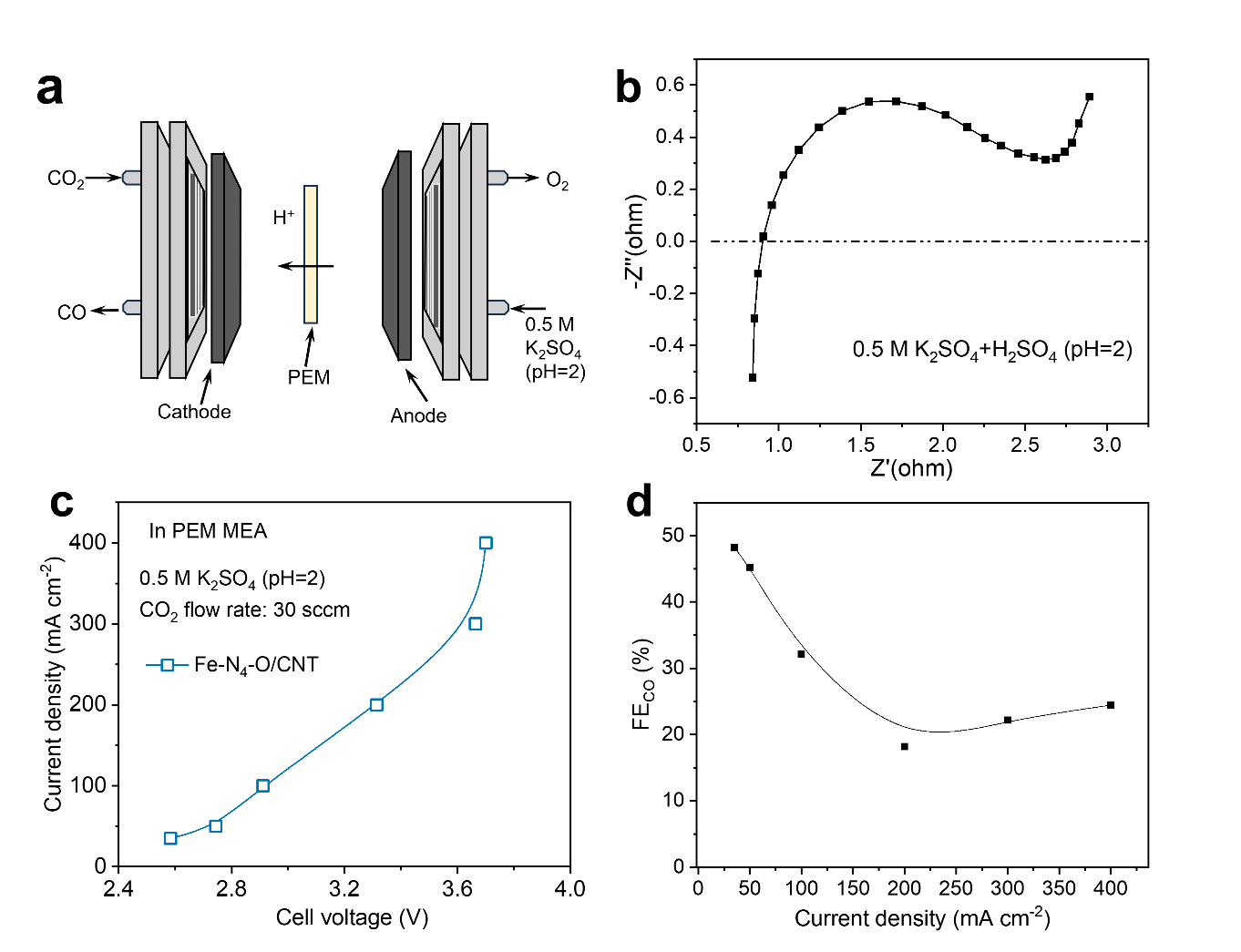


**Figure S34.** (a) Mechanistic diagram of acidic CO_2_RR performance testing in the PEM-MEA system. (b) Measured resistance of the PEM reactor with Fe-N_4_O/CNT as the cathode. (c) Cell voltage as a function of current density for CO_2_ electrolysis in 0.5 M K_2_SO_4_ + H_2_SO_4_ (pH=2). (d) FE_CO_ of Fe-N_4_O/CNT in the PEM reactor. Despite the presence of supporting salt in the anolyte, the locally strong acidity at the PEM membrane interface suppresses CO_2_RR on Fe-N_4_O/CNT, leading to poor CO performance in the PEM reactor.


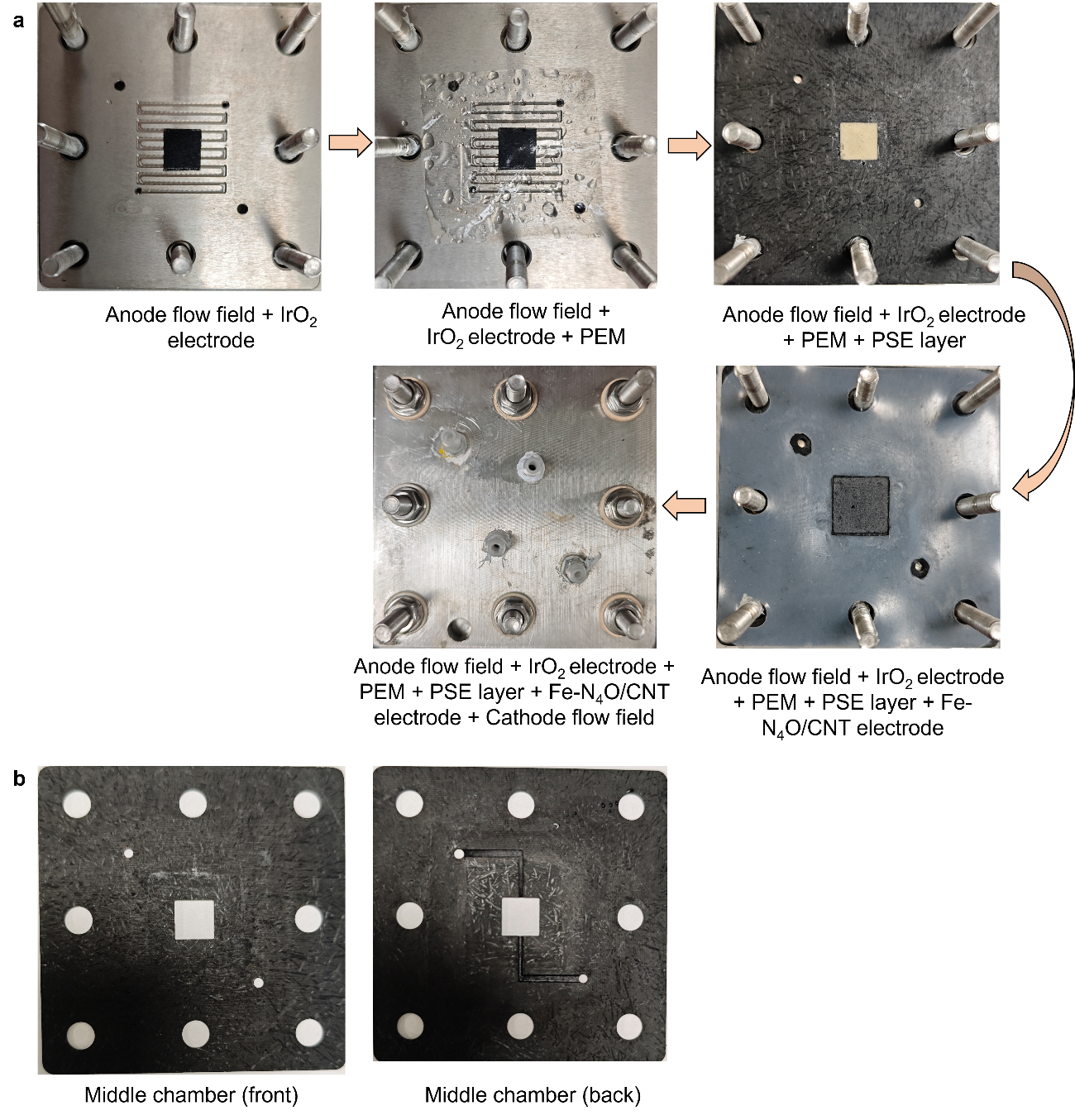


**Figure S35. (a)** Images illustrating the step-by-step process for assembling the PEM-PSE reactor. **(b)** Images showing the front and back of the middle chamber. As shown in Figure S35a, the PSE-PEM reactor was assembled as follows: Commercial iridium oxide was coated onto a titanium mesh about 0.25 mm thick to serve as the anode catalyst. A 0.25 mm silicone gasket was used, and the anode catalyst was placed in close contact with a Nafion 117 membrane. The intermediate chamber was then added, measuring 1.2 mm in thickness and with an active area of 1 × 1 cm^2^ (Figure S35b), featuring two inlets/outlets at the top and bottom. Next, the cathode was installed using Fe-N_4_O/CNT as the cathode catalyst. The cathode was cut to approximately 1.3 × 1.3 cm^2^ to seal the PSE-filled intermediate chamber and prevent PSE loss due to liquid flow during operation, which could cause voltage fluctuations.

**Figure S36.** Cell voltage response of the PEM reactor using Fe-N_4_O/CNT during stepwise current-density operation in 0.5 M K_2_SO_4_ + H_2_SO_4_ (pH=2). The voltage profiles at each current step show the dynamic evolution of cell voltage and stability under repeated current loading, and the steady-state values are used to construct the polarization behavior reported for this electrolyte condition.


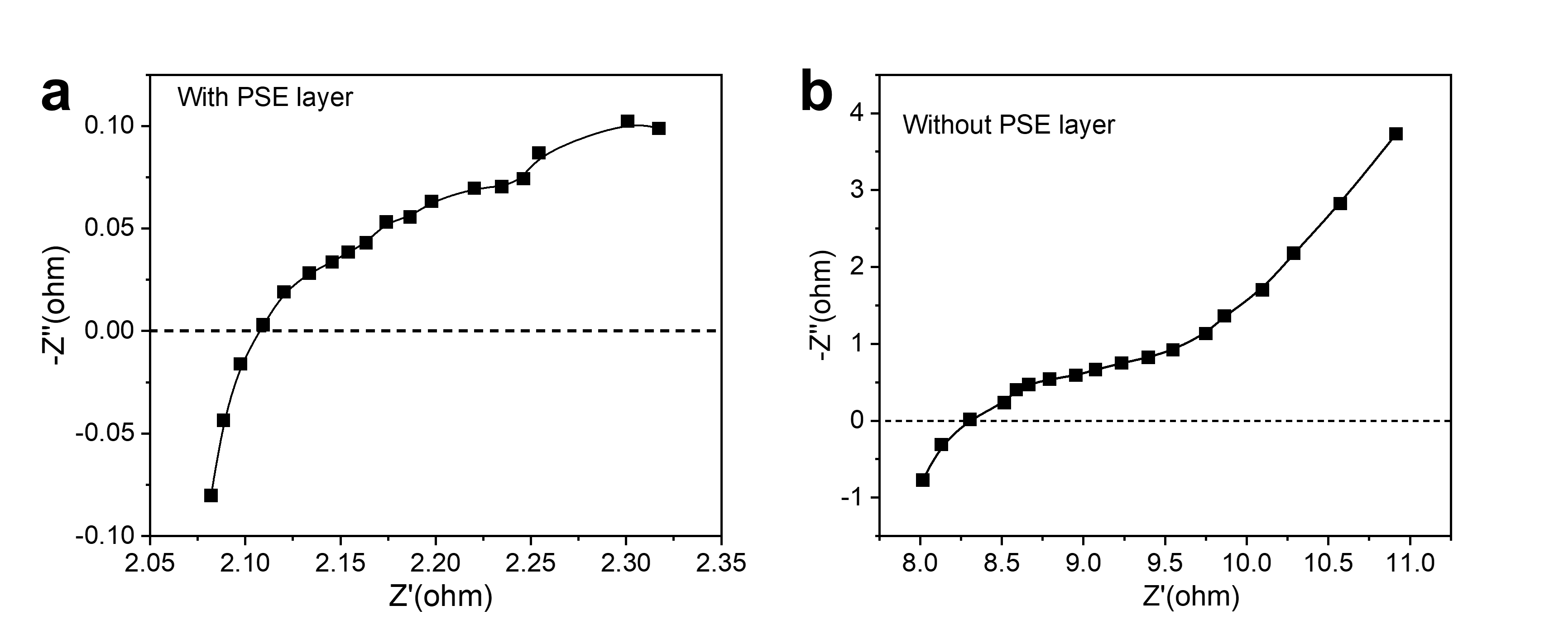


**Figure S37.** (a,b) Comparison of the reactor resistance measured with the porous solid-electrolyte (PSE) layer installed (a) and removed (b). The results quantify the contribution of the PSE layer to the overall ohmic resistance of the reactor and demonstrate how the PSE architecture affects ionic transport and interfacial contact resistance under identical measurement conditions.

**Figure S38.** CO production rate of Fe-N_4_O/CNT as a function of operating current density. The rate is derived from the CO formation current (*j*_CO_) and the measured FE_CO_, providing a direct measure of CO output under various electrolysis conditions.


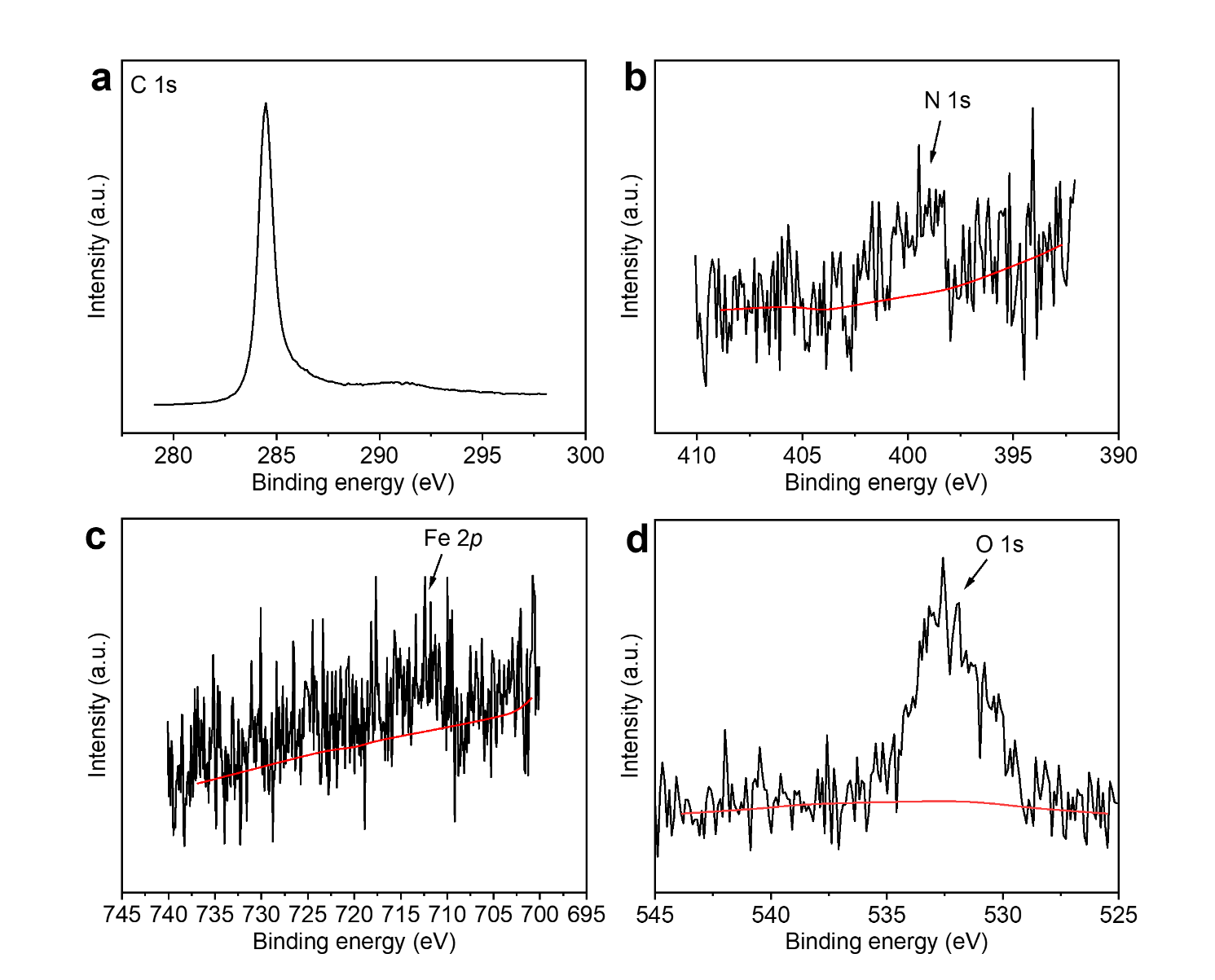


**Figure S39.** (a–d) High-resolution XPS spectra of Fe-N_4_O/CNT after the stability test. (a) C 1*s* spectrum showing the carbon environments of the CNT support. (b) N 1*s* spectrum reflecting the nitrogen species associated with the Fe-N_4_O motif and N-doped carbon. (c) Fe 2*p* spectrum confirming the retained Fe chemical state after prolonged operation. (d) O 1*s* spectrum showing oxygen-containing species, including Fe-O-related environments, after the stability test.


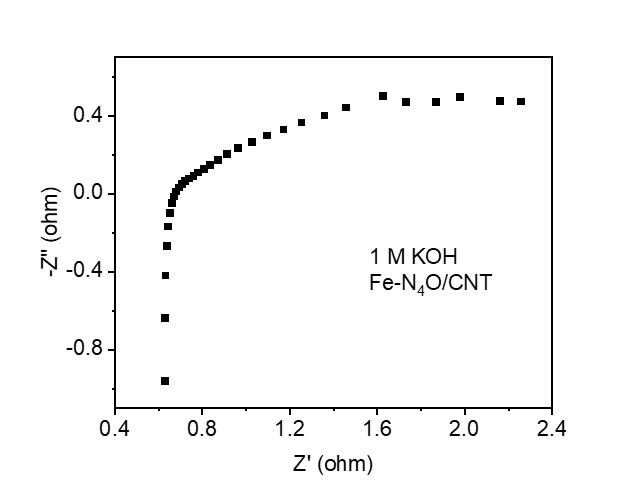


**Figure S40.** Resistance of the assembled MEA reactor measured in 1.0 M KOH under the same cell setup. The result indicates the overall ohmic contribution from the membrane, electrolyte, and interfacial contacts during alkaline operation.


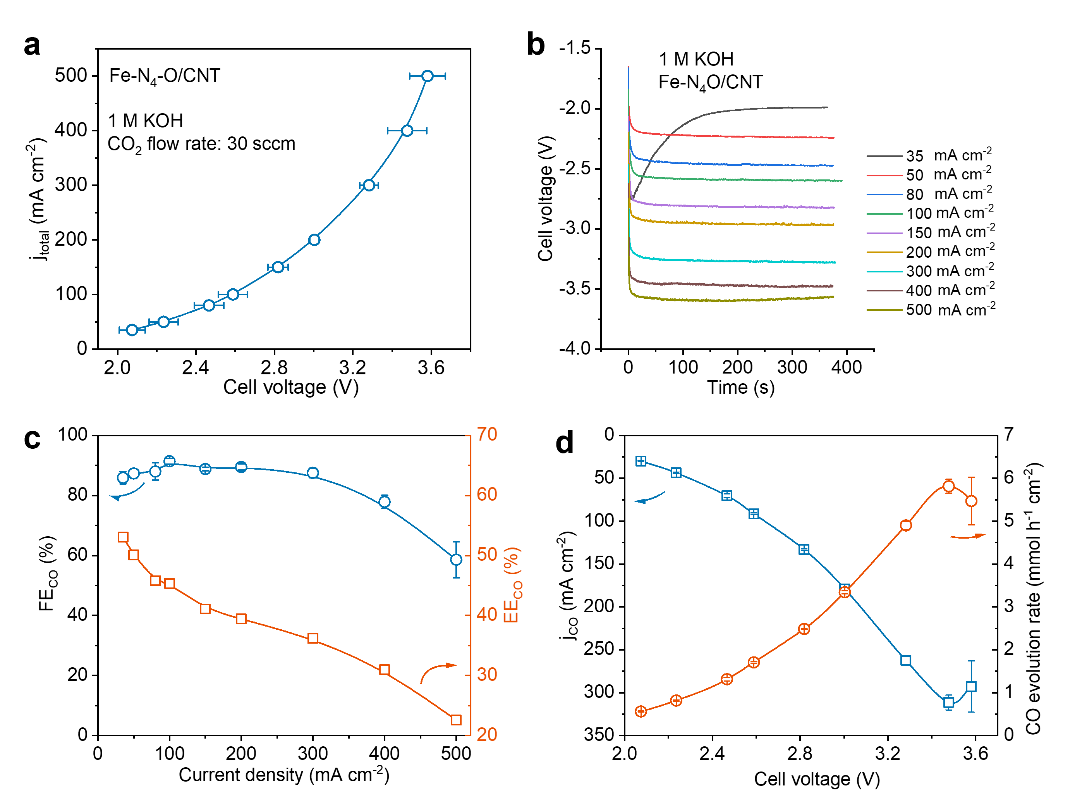


**Figure S41.** (a) Polarization (I–V) curves of Fe-N_4_O/CNT measured in 1.0 M KOH using the MEA configuration. (b) Corresponding cell voltage as a function of current density, derived from the steady-state values at each operating point. (c) Faradaic efficiency toward CO and energy efficiency (EE_CO_) of Fe-N_4_O/CNT in 1.0 M KOH at various current densities, where EE_CO_ is calculated from FE_CO_ and the measured cell voltage. (d) CO partial current density (*j*_CO_) and the related CO production rate derived from *j*_CO_, illustrating CO output as the current density increases.


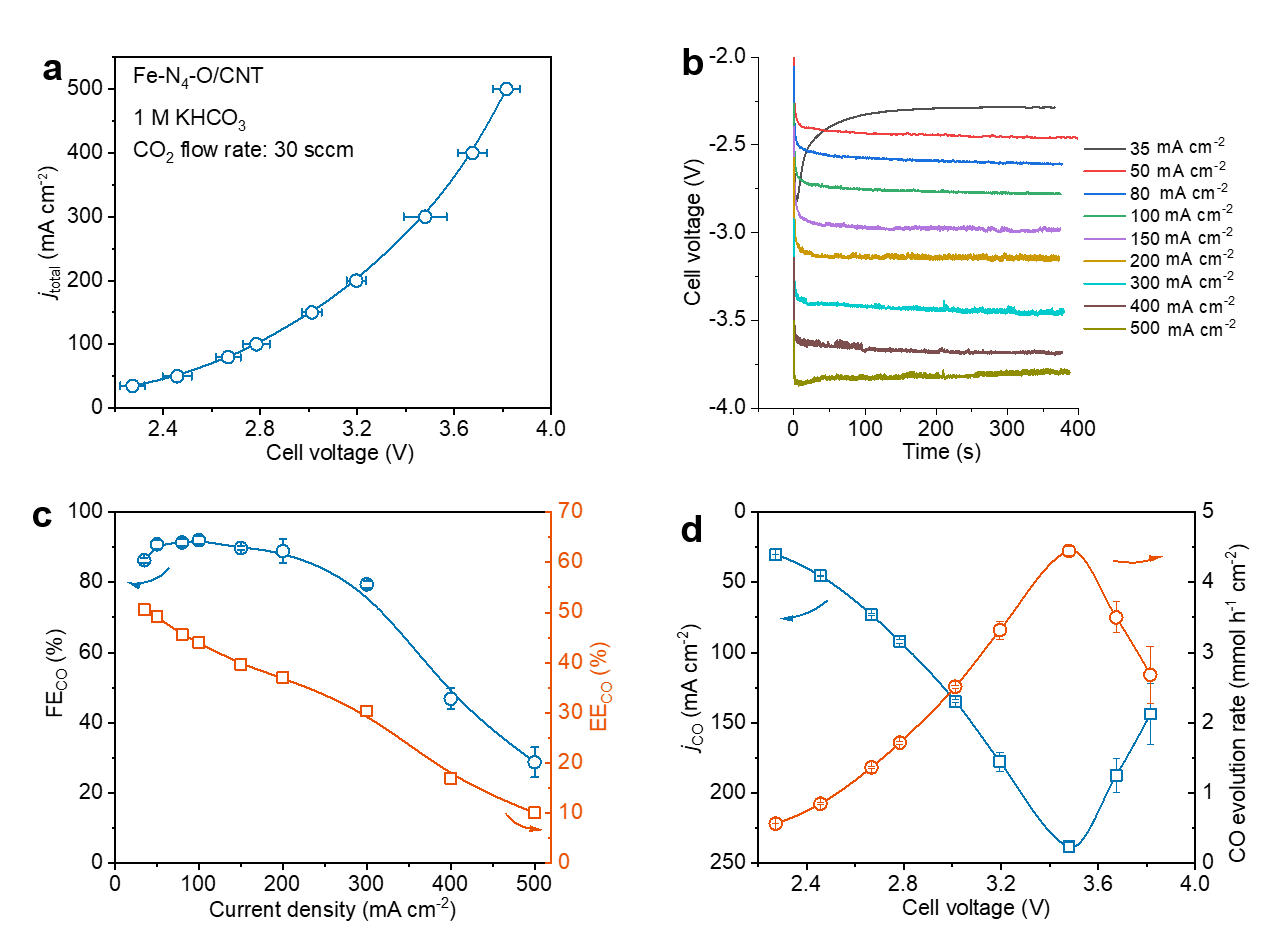


**Figure S42.** (a) Polarization (I–V) curves of Fe-N_4_O/CNT measured in 1.0 M KHCO_3_ using the MEA configuration. (b) Corresponding cell voltage as a function of current density, extracted from steady-state values at each operating point. (c) Faradaic efficiency toward CO and energy efficiency (EE_CO_) of Fe-N_4_O/CNT in 1.0 M KHCO_3_ at different current densities, where EE_CO_ is calculated from FE_CO_ and the measured cell voltage. (d) CO partial current density (*j*_CO_) and the corresponding CO production rate derived from *j*_CO_, showing CO output as the current density increases.


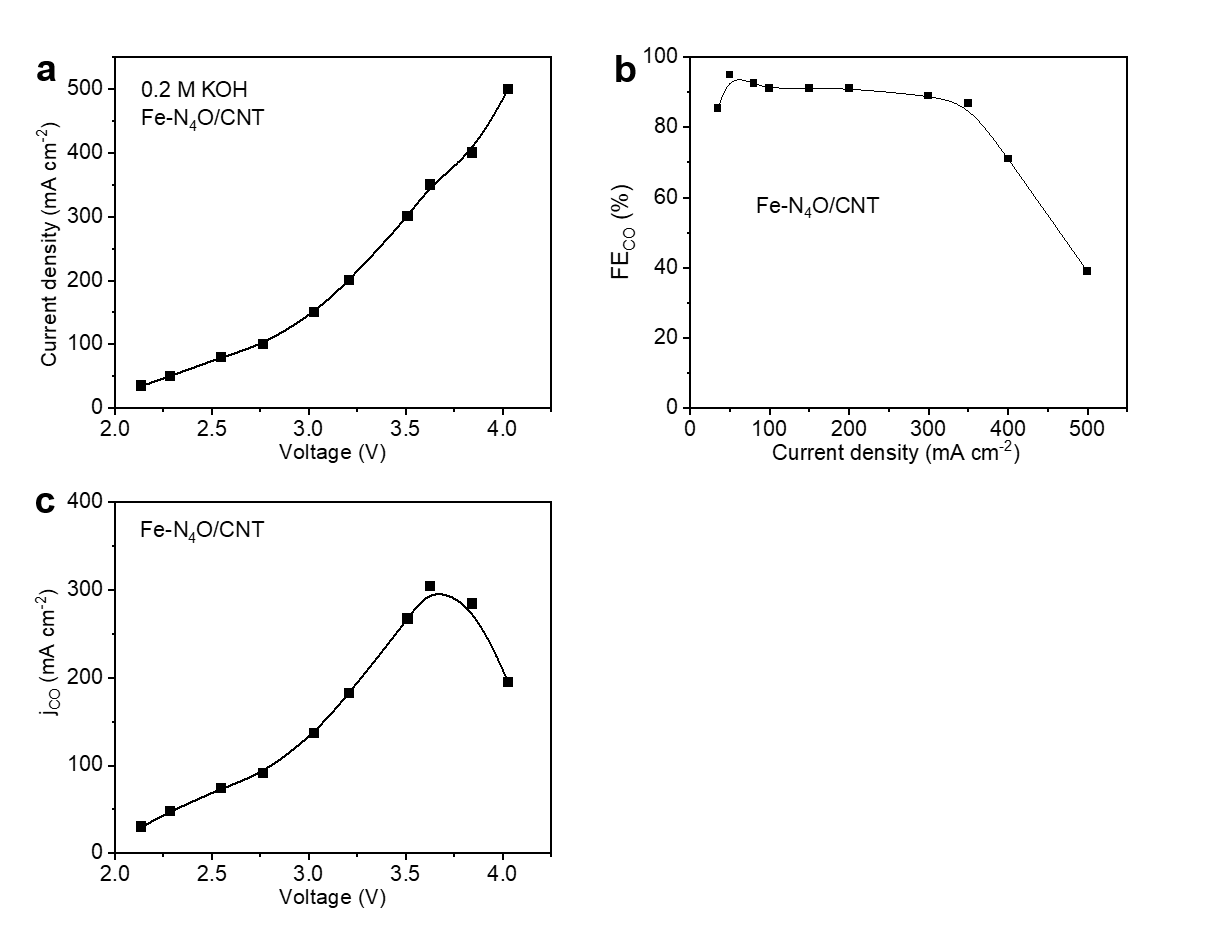


**Figure S43.** (a) Polarization (I–V) curves of Fe-N_4_O/CNT measured during CO_2_ electrolysis in 0.2 M KOH. (b) Faradaic efficiency toward CO for Fe-N_4_O/CNT at various operating points in 0.2 M KOH. (c) Partial current density for CO formation (j_CO_), calculated from the total current density and FE_CO_, illustrating the CO formation activity of Fe-N_4_O/CNT in 0.2 M KOH.

**Figure S44.** Stability assessment of Fe-N_4_O/CNT in the MEA reactor operated with 0.2 M KOH as the anolyte. The cell voltage is continuously monitored during long-term electrolysis, and FE_CO_ is periodically measured from the outlet gas to track CO selectivity over time.

**Figure S45.** Gas chromatography TCD traces of CO collected during CO_2_ electrolysis on Fe-N_4_O/CNT in the MEA reactor operated with 0.2 M KOH as the anolyte. The sequential chromatograms at different operation times show the evolution of the CO peak intensity during the stability test in 0.2 M KOH, which is used to quantify CO production and calculate FE_CO_.

**Figure S46.** Resistance of the PEM-PSE reactor with a reaction area of 6.25 cm^2^ measured under the same cell configuration. The value reflects the overall ohmic contribution from the PEM membrane, the PSE layer, and interfacial contacts in the integrated reactor.


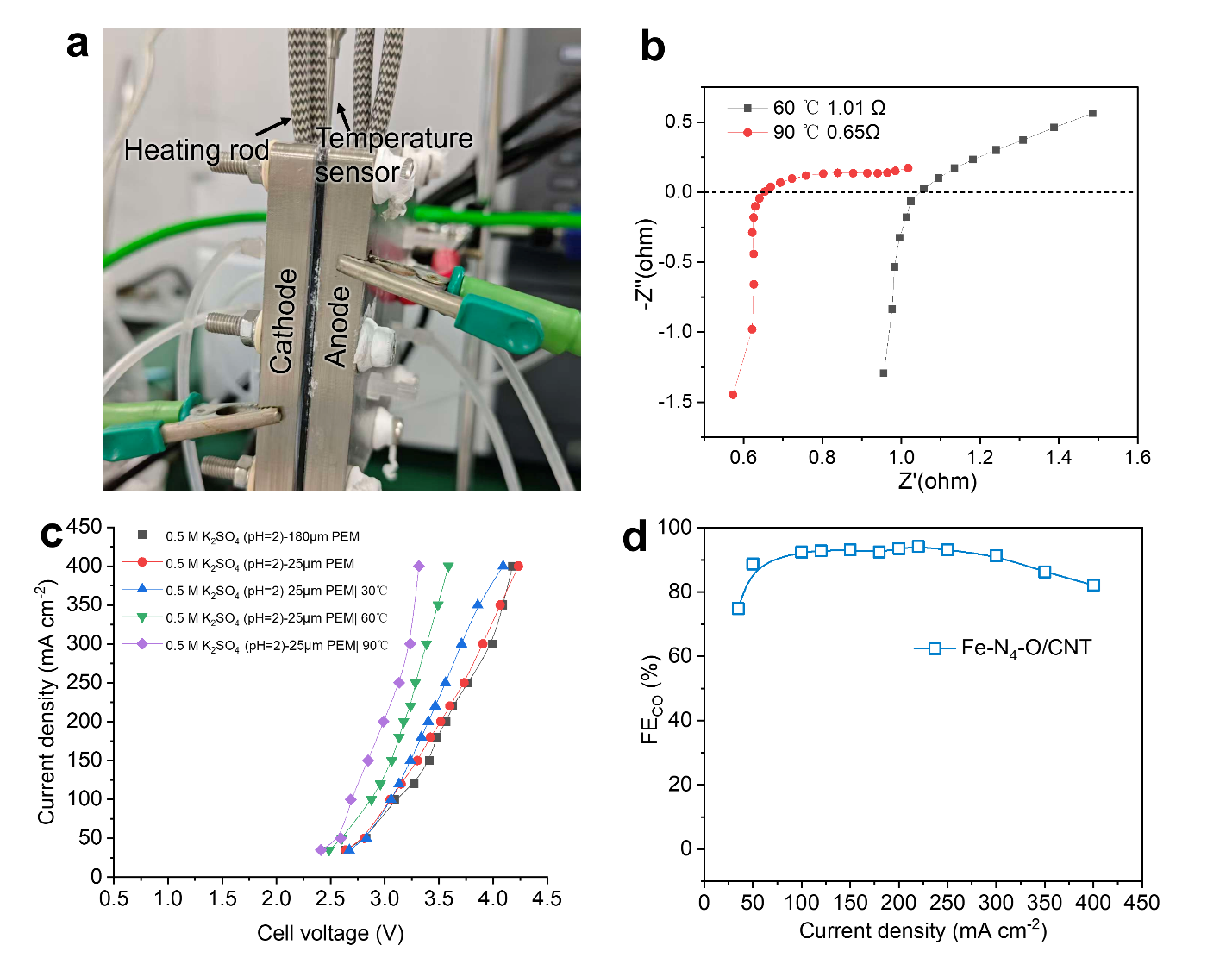


**Figure S47.** (a) Photograph of the heated PEM-PSE reactor, showing the integrated heating rod and temperature sensor near the cell body. (b) Resistance of the PEM-PSE reactor measured at different temperatures, demonstrating reduced ohmic losses with increasing operating temperature. (c) Polarization (I–V) behavior of the PEM-PSE reactor under optimized configurations, including a thinner PEM (180 μm versus 25 μm) and elevated operating temperatures (30 ℃, 60 ℃, and 90 ℃). (d) FE_CO_ of the PEM-PSE reactor as a function of current density using a PEM-25 μm, 60 ℃.

**Figure S48.** Experimentally fitted current–voltage relationship. We used current as the horizontal axis and voltage as the vertical axis to assess the nonlinear relationship between current and voltage. A quadratic equation was then constructed to analyze the relationship among current, voltage, and the cost of producing one tonne of CO.

**Figure S49.** Contour map of the total CO production cost for acidic CO_2_ electroreduction in the PSE-PEM reactor as a function of current density and FE_CO_, calculated using an electricity price of 0.02 USD per kWh. The dashed line indicates the CO market price (300 USD per ton), defining a profitable operating region where the modeled production cost is below this threshold. The symbols mark the operating window achieved in this work.

**3. Supplementary Table**

**Supplementary Table S1.** ICP-MS analysis results of Fe-N_4_/CNT and Fe-N_4_O/CNT (Fe wt.%)

| Sample | Fe (wt. %) | |
| --- | --- | --- |
| Fe-N_4_/CNT | | 0.50 |
| Fe-N_4_O/CNT | | 0.44 |

**Supplementary Table S2.** Spin-state analysis of Fe single atoms. Room temperature ^57^Fe Mössbauer parameters for Fe-N_4_O/CNT

| Sample | Peaks | IS (mm s^-1^) | QS (mm s^-1^) | Area (%) | Assignment |
| --- | --- | --- | --- | --- | --- |
| Fe-N_4_O/CNT | D_1_ | 0.183 | 1.062 | 84% | Intermediate-spin Fe |
|  | D_2_ | 0.363 | 0.643 | 16% | Low-spin Fe |

**Supplementary Table S3.** A comparison of reported Fe-based catalysts for CO current density.

| Catalyst | *j*_total_ (mA cm^-2^) | FE_CO_ | Electrolyte | *j_CO_* (mA cm^-2^) | Ref. |
| --- | --- | --- | --- | --- | --- |
| **Fe-N_4_O/CNT** | **400** | **~80.92** | 1 M KOH | **323.68** | **This work** |
| **Fe-N_4_O/CNT** | **350** | **~78.53** | 1 M KHCO_3_ | **274.85** | **This work** |
| **Fe-N_4_O/CNT** | **400** | **~83.79** | 0.5 M K_2_SO_4_ (pH=2) | **335.16** | **This work** |
| FeNC-1000 | 200 | ~85 | 0.05 M K_2_SO_4_ (pH=2) | 170 | [16] |
| Fe_1_-NSC | ~1 | ~98 | 0.5 M KHCO_3_ | 0.98 | [17] |
| Fe-S_1_N_3_ | ~88.67 | ~87 | 0.5 M KHCO_3_ | 77.14 | [18] |
| FePc/CNT | ~23.71 | ~97 | 0.5 M KHCO_3_ | 23 | [19] |
| HP-FeNC | ~5 | ~96 | 0.5 M KHCO_3_ | ~4.8 | [20] |
| p-Fe-N-C | ~6.1 | ~99 | 0.5 M KHCO_3_ | ~6.039 | [21] |
| Fe-poN-C/Fe | ~154.3 | ~95 | 1 M KOH | ~146.58 | [22] |
| Fe-N_4_O-C/Gr | ~20 | ~98.3 | 0.5 M KHCO_3_ | ~19.66 | [23] |
| Fe-NS-C | ~4.5 | ~89 | 0.1 M KHCO_3_ | ~4.005 | [24] |
| FeNC NSs-1000 | ~164 | ~90 | 1.0 M KOH | ~147.6 | [25] |
| FeN_5_@DNC | ~40 | ~98 | 0.5 M KHCO_3_ | ~39.2 | [26] |

**Supplementary Table S4.** A comparison of reported catalysts for the mass activity of the Fe site toward CO production.

| Catalyst | *j*_total_ (mA cm^-2^) | FE_CO_ | Electrolyte | *j_CO_* (A mg_Fe_^-1^) | Ref. |
| --- | --- | --- | --- | --- | --- |
| **Fe-N_4_O/CNT** | **400** | **~80.92** | 1 M KOH | **~74.16** | **This work** |
| **Fe-N_4_O/CNT** | **350** | **~78.53** | 1 M KHCO_3_ | **~62.47** | **This work** |
| **Fe-N_4_O/CNT** | **400** | **~83.79** | 0.5 M K_2_SO_4_ (pH=2) | **~76.17** | **This work** |
| FeNC-1000 | 200 | ~85 | 0.05 M K_2_SO_4_ (pH=2) | ~20.98 | [16] |
| Fe_1_-NSC | ~1 | ~98 | 0.5 M KHCO_3_ | ~0.11 | [17] |
| Fe-S_1_N_3_ | ~88.67 | ~87 | 0.5 M KHCO_3_ | ~7.49 | [18] |
| HP-FeNC | ~5 | ~96 | 0.5 M KHCO_3_ | ~0.51 | [20] |
| p-Fe-N-C | ~6.1 | ~99 | 0.5 M KHCO_3_ | ~0.66 | [21] |
| Fe-poN-C/Fe | ~154.3 | ~95 | 1 M KOH | ~12.86 | [22] |
| Fe-N_4_O-C/Gr | ~20 | ~98.3 | 0.5 M KHCO_3_ | ~2.1 | [23] |
| Fe-NS-C | ~4.5 | ~89 | 0.1 M KHCO_3_ | ~1.1 | [24] |
| FeN_5_@DNC | ~40 | ~98 | 0.5 M KHCO_3_ | ~4.56 | [26] |
| Fe-P-N-C | ~600 | ~97 | 1.0 M KOH | ~34.73 | [27] |

**Supplementary Table S5.** Preliminary TEA baseline parameters for acid CO_2_RR system.

| **Parameters/method** | Acid CO_2_RR system |
| --- | --- |
| Current density (mA cm^-2^) | 100 |
| FE_CO_ (%) | 90 |
| FE_H2_ (%) | 9.41 |
| Cell voltage (V) | 3.09 |
| Conversion rate (%) | 80.97 |
| Electricity price ($ kWh^-1^) | 0.03 |
| Cathode products production (CO, kg/day) | 100,000 |
| Ref. electrolyzer cost ($ m^-2^) | 919.7 |
| H_2_O price ($ gal^-1^) | 0.0054 |
| CO_2_ cost ($ ton^-1^) | 40 |
| CO market value ($ ton^-1^) | 300 |

**Supplementary Table S6** EXAFS fitting parameters at the Fe K-edge for various samples（*Ѕ*_0_^2^=0.95）

| Sample | Shell | *CN^a^* | *R*(Å)*^b^* | *σ*^2^(×10^-3^ Å^2^)*^c^* | Δ*E*_0_(eV)*^d^* | *R* factor |
| --- | --- | --- | --- | --- | --- | --- |
| Fe-N_4_O/CNT | Fe-O | 1.3 | 1.83 | 3.0 | 0.51 | 0.02 |
|  | Fe-N | 4.3 | 2.03 | 2.5 | -5.51 |  |

*^a^CN*, coordination number; *^b^R*, distance between the absorber and backscatter atoms; *^c^σ*^2^, Debye-Waller factor accounting for both thermal and structural disorders; *^d^ΔE*_0_, inner potential correction; the *R* factor indicates the goodness of the fit. *S*0^2^ was fixed at 0.95, according to the experimental EXAFS fit of Fe foil by fixing CN as the known crystallographic value. Fitting range: 3.5 ≤ *k* (/Å) ≤ 10.0 and 1.0 ≤ *R* (Å) ≤ 3.0 (Fe-N_4_O/CNT). A reasonable range of EXAFS fitting parameters: 0.700 < *Ѕ*_0_^2^ < 1.000; *CN >* 0; *σ*^2^ > 0 Å^2^; |Δ*E*_0_| < 10 eV; *R* factor < 0.02.

Data reduction, analysis, and EXAFS fitting were performed using the Athena and Artemis programs from the Demeter data analysis suite [1], which employs the FEFF6 program [2] to fit the EXAFS data. The sample's energy calibration was carried out with a standard Fe foil, which was measured simultaneously as a reference. A linear function was subtracted from the pre-edge region, and then the edge jump was normalized using Athena software. The k^3^-weighted χ(k) data were Fourier transformed after applying a Hanning window function (Δk = 1.0) in Fe foil. For Fe-N_4_O/CNT, the k^3^-weighted χ(k) data were Fourier transformed after applying a Sine window function (Δk = 1.0). In EXAFS modeling, the global amplitude parameters (*CN, R, σ*^2,^ and Δ*E*_0_) were obtained through nonlinear least-squares fitting of the EXAFS equation to the Fourier-transformed data in R-space using Artemis software. The EXAFS of the Fe foil was fitted, and the resulting amplitude reduction factor *S_0_^2^* (0.95) was used in subsequent analyses to determine the coordination numbers (CNs) for the Fe-N and Fe-O scattering paths in the sample. The corresponding XANES spectra of Fe-N_4_O/CNT were fitted using the finite difference method provided by the FDMNES code.

**Reference**

1. B. Ravel, M. Newville, *J. Synchrotron Radiat.* **2005**, *12*, 537.
2. S. Zabinsky, J. Rehr, A. Ankudinov, *Phys. Rev. B* **1995**, *52*, 2995.
3. G. Kresse, J. Furthmüller, *Phys. Rev. B* **1996**, *54*, 11169–11186.
4. G. Kresse, D. Joubert, *Phys. Rev. B* **1999**, *59*, 1758.
5. J. Perdew, K. Burke, M. Ernzerhof, *Phys. Rev. Lett.* **1996**, *77*, 3865.
6. S. Grimme, J. Antony, S. Ehrlich, H. Krieg, *J. Chem. Phys.* **2010**, *132*, 154104.
7. W. Tang, E. Sanville, G. Henkelman, *J. Phys.: Condens. Matter* **2009**, *21*, 084204.
8. S. Maintz, V. Deringer, A. Tchougréeff, R. Dronskowski, *J. Comput. Chem.* **2016**, *37*, 1030.
9. J. Nørskov, J. Rossmeisl, A. Logadottir, L. Lindqvist, J. Kitchin, T. Bligaard, H. Jonsson, *J. Phys. Chem. B* **2004**, *108*, 17886.
10. K. Mathew, R. Sundararaman, K. Letchworth-Weaver, T. Arias, R. Hennig, *J. Chem. Phys.* **2014**, *140*, 084106.
11. S. Nosé, *J. Chem. Phys.* **1984**, *81*, 511–519.
12. W. G. Hoover, *Phys. Rev. A* **1985**, *31*, 1695–1697.
13. X. Wang, P. Ou, A. Ozden, S. Hung, J. Tam, C. M. Gabardo, J. Y. Howe, J. Sisler, K. Bertens, F. P. G. de Arquer, R. K. Miao, C. P. O’Brien, Z. Wang, J. Abed, A. S. Rasouli, M. Sun, A. H. Ip, D. Sinton, E. H. Sargent, *Nat. Energy* **2022**, *7*, 170–176.
14. H. Shin, K. Hansen, F. Jiao, *Nat. Sustain.* **2021**, *4*, 911.
15. X. Li, C. Hao, S. Luo, A. Yu, P. Li, B. Xiong, Z. Fu. J. Zhu Y. Seow, Y. Lu, X. Wang, T. Ma, Z. J. Xu, Z. Cheng. *J. Energy Chem.* **2026**, *114*,116-125.
16. Tang, Q. Hao, J. Wu, Y. Zhang, P. Sun, D. Wang, C. Tian, H. Zhong, Y. Zhu, K. Huang, K. Liu, X. Zhang, J. Lu, *Adv. Energy Mater.* **2024**, *14*, 2401364.
17. S. Chen, X. Li, C. W. Kao, T. Luo, K. Chen, J. Fu, C. Ma, H. Li, M. Li, T. S. Chan, M. Liu, *Angew. Chem. Int. Ed.* **2022**, *61*, e202206233.
18. Z. Jin, D. Jiao, Y. Dong, L. Liu, J. Fan, M. Gong, X. Ma, Y. Wang, W. Zhang, L. Zhang, Z. G. Yu, D. Voiry, W. Zheng, X. Cui, *Angew. Chem. Int. Ed.* **2024**, *136*, e202318246.
19. Y. Zeng, J. Zhao, S. Wang, X. Ren, Y. Tan, Y. Lu, S. Xi, J. Wang, F. Jaouen, X. Li, Y. Huang, T. Zhang, B. Liu, *J. Am. Chem. Soc.* **2023**, *145*, 15600.
20. C. Jia, Y. Zhao, S. Song, Q. Sun, Q. Meyer, S. Liu, Y. Shen, C. Zhao, *Adv. Energy Mater.* **2023**, *13*, 2302007.
21. Y. Zhao, Z. Shi, F. Li, C. Jia, Q. Sun, Z. Su, C. Zhao, *ACS Catal.* **2024**, *14*, 3926.
22. C. Wang, X. Wang, H. Ren, Y. Zhang, X. Zhou, J. Wang, Q. Guan, Y. Liu, W. Li, *Nat. Commun.* **2023**, *14*, 5108.
23. S. Chen, J. Chen, Y. Li, S. Tan, X. Liao, T. Zhao, K. Zhang, E. Hu, F. Cheng, H. Wang, *Adv. Funct. Mater.* **2023**, *33*, 2300801.
24. F. Pan, B. Li, E. Sarnell, S. Hwang, Y. Gan, X. Feng, X. Xiang, N. M. Adli, T. Li, D. Su, G. Wu, G. Wang, Y. Li, *Nano Energy* **2020**, *68*, 104384.
25. D. Lin, T. Wang, Z. Zhao, Y. Liu, H. Song, X. Yan, Z. Li, S. Yao, X. Hu, L. Lei, B. Yang, Y. Hou, *Nano Energy* **2023**, *113*, 108568.
26. Y. Bao, J. Xiao, Y. Huang, Y. Li, S. Yao, M. Qiu, X. Yang, L. Lei, Z. Li, Y. Hou, G. Wu, B. Yan, *Angew. Chem. Int. Ed.* **2024**, *63*, e202406030.
27. Y. Zang, Y. Liu, R. Lu, Q. Yang, B. Wang, M. Zhang, Y. Mao, Z. Wang, Y. Lum, Adv. Mater. **2025**, 37, 2417034.
